# Number of Influenza Risk Factors Informs an Adult's Increased Potential of Severe Influenza Outcomes: A Multiseason Cohort Study From 2015 to 2020

**DOI:** 10.1093/ofid/ofae203

**Published:** 2024-04-12

**Authors:** Ian McGovern, Katherine Cappell, Alina N Bogdanov, Mendel D M Haag

**Affiliations:** CSL Seqirus, Center for Outcomes Research and Epidemiology, Waltham, Massachusetts, USA; Veradigm, Chicago, Illinois, USA; Veradigm, Chicago, Illinois, USA; CSL Seqirus, Center for Outcomes Research and Epidemiology, Amsterdam, the Netherlands

**Keywords:** adult, comorbidity, hospitalization, influenza, risk

## Abstract

**Background:**

While studies have evaluated factors influencing the risk of severe influenza outcomes, there is limited evidence on the additive impact of having multiple influenza risk factors and how this varies by age.

**Methods:**

Patients ≥18 years of age in the United States were evaluated retrospectively in 5 seasonal cohorts during the 2015–2020 influenza seasons. Patient-level electronic medical records linked to pharmacy and medical claims were used to ascertain covariates and outcomes. Multivariable logistic regression models were fitted for the overall population and by age subgroups to evaluate the association of demographic and clinical characteristics with odds of influenza-related medical encounters (*ICD-10* codes J09*–J11*). The logistic regression models included sex, race/ethnicity, geographic region, baseline health care resource use, vaccination status, specific high-risk comorbidities, number of influenza risk factors, body mass index, and smoking status. Odds ratios from each of the 5 seasons were summarized via fixed effect meta-analysis.

**Results:**

Season cohort sizes ranged from 887 260 to 3 628 168 adults. Of all patient characteristics evaluated, an individual’s cumulative number of high-risk influenza conditions, as defined per the Centers for Disease Control and Prevention, was the most predictive of an increased probability of having an influenza-related medical encounter overall and across age groups. For adults of any age, odds ratios for influenza hospitalization ranged from 1.8 (95% CI, 1.7–2.0) for 1 risk factor to 6.4 (95% CI, 5.8–7.0) for ≥4 risk factors.

**Conclusions:**

These results show that a simple measure such as the number of influenza risk factors can be highly informative of an adult's potential for severe influenza outcomes.

## Teaser

This descriptive study evaluated the association of patient characteristics and influenza-related medical encounters and found that a simple measure such as the number of influenza risk factors can be highly informative of an adult's potential for severe influenza outcomes.

Seasonal influenza presents a heavy burden to the US health care system, and in the 5 seasons between 2015 and 2020, it was estimated to be the cause of 11 to 21 million medical visits, 280 000 to 710 000 hospitalizations, and 23 000 to 52 000 deaths annually [1]. Certain subgroups of the population are at particular risk of severe outcomes due to an influenza infection. Among adults, the US Centers for Disease Control and Prevention (CDC) defines these risk factors as age ≥65 years, specific high-risk medical conditions, certain racial and ethnic backgrounds (Black, Hispanic, and American Indian and Alaska Native), pregnancy, and living in a residential care setting [2, 3]. While several studies have evaluated how older age and the presence of at least 1 high-risk condition or specific risk factor influences the risk of severe influenza outcomes [4–7], there is limited evidence on the potential additive impact of having multiple high-risk conditions and how this effect may vary by age [8]. Identifying which individuals are at increased risk of influenza can be informative for individual- and program-level decisions on influenza vaccination. Evaluating the potential additive impact of having multiple risk factors further improves the ability to identify those most at risk and thus can result in more informed decision making.

This study assessed how the number of CDC-defined high-risk conditions was associated with the odds of having an influenza outpatient visit, emergency department (ED) visit, or hospitalization in the overall adult population and among age subgroups. In addition, this study evaluated how select demographic and clinical characteristics are associated with the odds of medically attended influenza in the overall adult population and among age subgroups.

## METHODS

### Study Design

We conducted a retrospective observational cohort study evaluating risk factors related to the odds of having an influenza-related medical encounter (IRME). IRMEs included influenza-related outpatient visits (eg, general practitioner visit), ED visits, or hospitalizations. Risk factors for IRMEs were assessed in 5 independent retrospective seasonal cohorts with data from 5 influenza seasons: 2015–2016, 2016–2017, 2017–2018, 2018–2019, and 2019–2020.

### Data Sources

This study leveraged an integrated data set of outpatient electronic health records (EHRs) linked to closed medical and pharmacy claims data. This data set included US outpatient EHR data from the Veradigm Network EHR, with outpatient, inpatient, and pharmacy claims data from Komodo Health [9]. The data for the 2015–2016 season may be incomplete because while enrollment data were available prior to 2015, claims data prior to 1 January 2015 were not.

### Study Population

Each of the 5 influenza seasons was treated as a separate retrospective cohort, with cohort selection, baseline characteristic assessment, and outcome assessment occurring independently for each season. Hence, individuals potentially contributed to 1 or more of the seasonal assessments.

This study included all adults (≥18 years old) at the start of the influenza season who met the following criteria. First, they had EHR activity at least 1 year prior to the start of the influenza season and during the influenza season. An influenza season was defined as week 40 of 1 calendar year through week 20 in the subsequent calendar year. In the 2019–2020 season, we truncated the influenza season at the end of week 10 (7 March 2020) to avoid complications in the analysis due to the potential widespread circulation of the SARS-CoV-2 virus. Second, individuals had continuous claims enrollment from 1 year prior to the start of each influenza season to at least 120 days after the end of each influenza season. We excluded those with missing gender or geographic data and those who had an IRME during the off-season period (ie, during the 19-week period between the end of the previous influenza season and the start of the subsequent influenza season). Adults who met the criteria were stratified by age at the start of the season into 1 of 3 categories (18–49, 50–64, and ≥65 years).

### Explanatory Variables

Demographics and medical history were evaluated during a 1-year baseline period that spanned epidemiology week 40 in the calendar year preceding the start of the influenza season through week 39, just before the start of the influenza season (Supplementary Figure 1). For each patient, we captured the following demographic characteristics: age at the start of the influenza season, sex, race (Asian, Black, White, other, unknown), ethnicity (Hispanic or non-Hispanic), and geographic region (Northeast, Midwest, South, West, unknown). We evaluated several measures of health care resource use during the off-season period, including the number of individuals with an outpatient visit and the number of outpatient visits, the number with an ED visit and the number of ED visits, and the number with a hospitalization and the number of hospitalizations. Vaccination status was captured if it occurred during the influenza season and was recorded in outpatient or claims records. Although vaccination status was a covariate in all of the models, vaccine effectiveness was not evaluated because the vaccine coverage levels in this data set, as compared with those reported by the CDC, suggest that there may be a high proportion of individuals with missing influenza vaccination data—potentially due to vaccinations that occur outside a medical care setting, such as on-site work vaccination clinics. Due to this misclassification of vaccine exposure status, vaccine effectiveness would likely bias toward the null.

Clinical characteristics were the Charlson Comorbidity Index [10], measures of baseline cardiovascular risk (cardiac hospitalizations during the baseline period), smoking history, and CDC-defined influenza risk factors that increase one’s risk of severe outcomes following an influenza infection [3], as listed in Supplementary Table 1. The CDC-defined influenza risk factors are asthma, neurologic and neurodevelopmental conditions, blood disorders, chronic lung disease, endocrine disorders (eg, diabetes), heart disease, kidney diseases, liver diseases, metabolic disorders (eg, inherited metabolic disorders, mitochondrial disorders), obesity (body mass index [BMI] ≥40), weakened immune system due to disease or treatment, and stroke. Patients were considered to have a given high-risk condition if they had 1 or more diagnoses consistent with that disease during the baseline period (based on *ICD-10* codes). The CDC considers only those with a BMI ≥40 to at higher risk, although the upper cutoff for BMI in this study was ≥30 due to the limited number with a BMI ≥40. Although age ≥65 years is considered a risk factor by the CDC, it was not a risk factor in this analysis to improve comparability with other age groups. Other CDC-defined influenza risk factors, such as certain racial and ethnic backgrounds, pregnancy, and living in a residential care setting, were not included due to insufficient data to accurately assess their presence. While race and ethnicity were included in the study, they were not in the risk factor count because the race categories in the database did not allow us to identify those who were American Indian or Alaskan Native (which were 2 of the 3 CDC-defined high risk racial groups, with Black being the third) and 15% to 30% had missing race information.

For the assessment of BMI on odds of having an IRME, healthy weight individuals (BMI, 18.5–24.9) were compared with those who were underweight (<18.5), overweight (25.0–29.9), and obese (≥30). For smoking history, patients who never smoked were compared with current and former smokers (among those with a smoking history recorded in their EHRs).

In addition to documenting the presence of CDC-defined risk factors, we calculated the total number of CDC-defined risk factors that an individual had. For each season of eligibility, patients were classified as having 0, 1, 2, 3, or ≥4 risk factors based on the number of CDC-defined high-risk condition diagnoses in the baseline period for that season. The number of risk factors was capped at ≥4 based on a preliminary assessment that found relatively few individuals with ≥5 risk factors. If an individual had >1 diagnosis in a single risk factor category (eg, 2 diagnoses related to liver disease), that was still considered to indicate the presence of only a single risk factor. Demographic and clinical characteristics other than the CDC-defined high-risk conditions did not contribute toward the number of risk factors that one had.

### Outcome Variables

The outcomes of interest were influenza-related outpatient visits, ED visits, or hospitalizations. Influenza-related outpatient visits were identified by EHR and claims data. Because the underlying EHR data were limited to the outpatient setting, influenza-related ED visits and inpatient hospitalizations were identified from claims only. We considered ED visits that occurred on the same day or on the day preceding a hospitalization to be part of the hospitalization. The codes that we used to identify influenza diagnosis were *ICD-9-CM* codes 487.x and 488.x and *ICD-10* codes J09^∗^, J10^∗^, and J11^∗^.

### Data Analysis

Cohort characteristics are summarized descriptively by counts and percentages for categorical variables and by mean and SD for continuous variables. Median and IQR are reported for select continuous variables. We assessed differences in baseline characteristics between patients aged 50 to 64 years and 18 to 49 or ≥65 years using standardized mean differences. Standardized mean differences >|0.1| were considered meaningful differences.

The association of explanatory variables with IRMEs was evaluated in overall age groups and age-stratified logistic regression models. Due to the large number of effect estimates generated, as a post hoc analysis, odds ratios from the 5 seasons were summarized by fixed effect meta-analysis to better understand general trends across the seasons. A fixed effect model was used, rather than a random effects model, because it was assumed that, despite season-to-season variation in the severity of influenza seasons, any given risk factor would likely convey a more fixed proportionate impact on risk of a severe outcome following an influenza infection.

Descriptive statistics and logistic regression models were generated by SAS version 9.4 (SAS Institute). R version 4.2.2 (R Foundation for Statistical Computing) was used for data visualization and post hoc meta-analyses with the package *meta* [11].

### Quality and Ethics

The study was designed, implemented, and reported in accordance with good pharmacoepidemiological practice, applicable local regulations, and the ethical principles laid down in the Declaration of Helsinki [12, 13]. The study findings have been reported according to the RECORD recommendations (Reporting of Studies Conducted Using Observational Routinely Collected Health Data) [14]. This linked EHR and claims data set has been certified as statistically deidentified through a formal determination by a qualified expert as defined in §164.514(b)(1) of the privacy rule per the Health Insurance Portability and Accountability Act of 1996.

## RESULTS

The final study population comprised 887 260 adults in the 2015–2016 season, 2 789 372 in the 2016–2017 season, 3 202 455 in the 2017–2018 season, 3 628 168 in the 2018–2019 season, and 3 310 936 in the 2019–2020 season (Supplementary Table 2). Within these populations, 37.8% to 44.3% were in the 18- to 49-year-old cohort, 33.5% to 38.6% in the 50- to 64-year-old cohort, and 17.2% to 28.7% in the ≥65-year-old cohort across seasons.

Baseline characteristics are reported by season and age group in Supplementary Table 3. In all seasons, there tended to be a higher percentage of females in the cohort aged 18 to 49 years vs 50 to 64 years (standardized mean differences, −0.130 to −0.111). Among those 18 to 49 years old, a median 63.6% were female, as compared with 57.5% and 57.2% among those 50 to 64 and ≥65 years old, respectively. The majority in all seasons and age groups were White and non-Hispanic.

**Figure 1.**
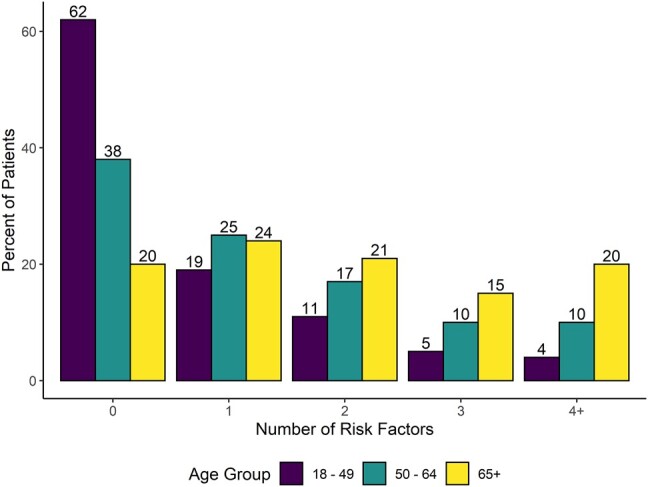
Median percentage of patients with high-risk conditions in seasons 2015–2016 through 2019–2020 by age group.

The majority of patients aged 50 years had high-risk conditions (Figure 1). The percentage with at least 1 risk factor increased with age from a median 37.7% among those 18 to 49 years old to 61.5% for those 50 to 64 years old to 79.7% among those ≥65 years old (Supplementary Table 3). The percentage with at least 4 high-risk conditions increased with age from a median 3.7% to 9.9% and 20.4%, respectively. Other measures indicating that baseline health status decreased with age were greater use of health care services in the outpatient setting during the off-season period, higher Charlson Comorbidity Index, and higher prevalence of specific high-risk comorbidities.

### Association Between the Cumulative Number of CDC-Defined Risk Factors and IRME

All results are from the meta-analysis and reported as odds ratios (ORs) with 95% CIs. Unless otherwise stated, all reported odds ratios are for the overall population of adults (≥18 years old).

**Figure 2.**
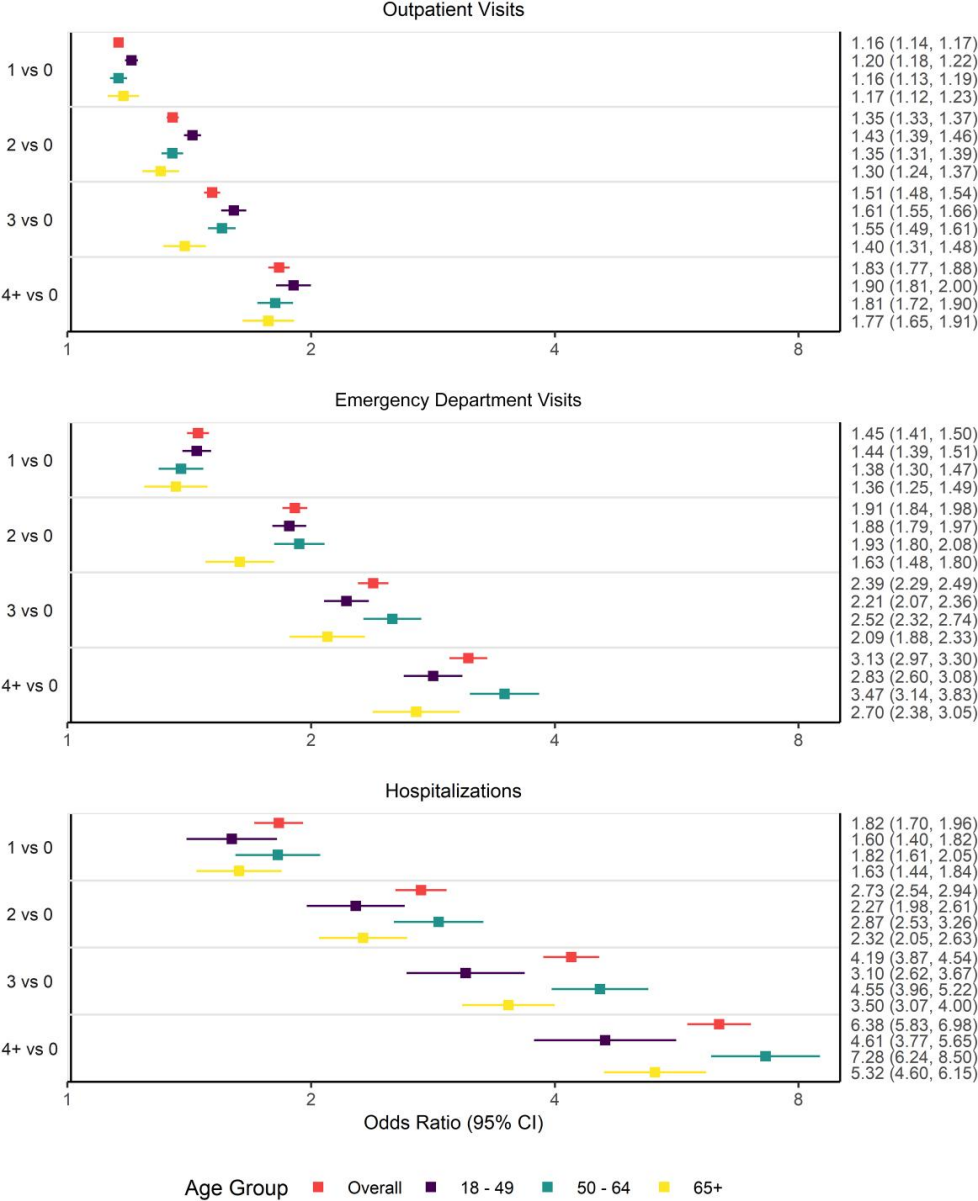
Number of high-risk conditions as a risk factor for influenza-related outpatient visits, emergency department visits, and hospitalizations.

In the overall population, individuals with conditions that were CDC-defined risk factors had higher odds of having an influenza-related outpatient visit, ED visit, or hospitalization as compared with those having zero high-risk conditions (Figure 2). In addition, the odds of having an influenza-related outpatient visit increased with the number of risk factors (1 condition: OR, 1.16 [95% CI, 1.14–1.17]; 2 conditions, 1.35 [1.33–1.37]; 3 conditions, 1.51 [1.48–1.54]; ≥4 conditions, 1.83 [1.77–1.88]). The trend of the odds rising with every added risk factor was also observed for influenza-related ED visits (1–4 conditions: OR, 1.45 [95% CI, 1.41–1.50]; 1.91 [1.84–1.98]; 2.39 [2.29–2.49]; 3.13 [2.97–3.30]) and hospitalizations (1.82 [1.70–1.96], 2.73 [2.54–2.94], 4.19 [3.87–4.54], 6.38 [5.83–6.98]).

The same trend of an increase in odds of IRMEs with a rising number of risk factors was observed in each age group (Figure 2). The effect of a rising number of risk factors on the increasing odds of influenza-related ED visits and hospitalizations was most pronounced for individuals aged 50 to 64 years. Notably, the differences across age groups with the same number of risk factors were smaller than the differences across risk factor groups of the same age. This suggests that the number of CDC-defined clinical risk factors is a more significant driver of IRME than age.

### Nonclinical Risk Factors for IRME

**Figure 3.**
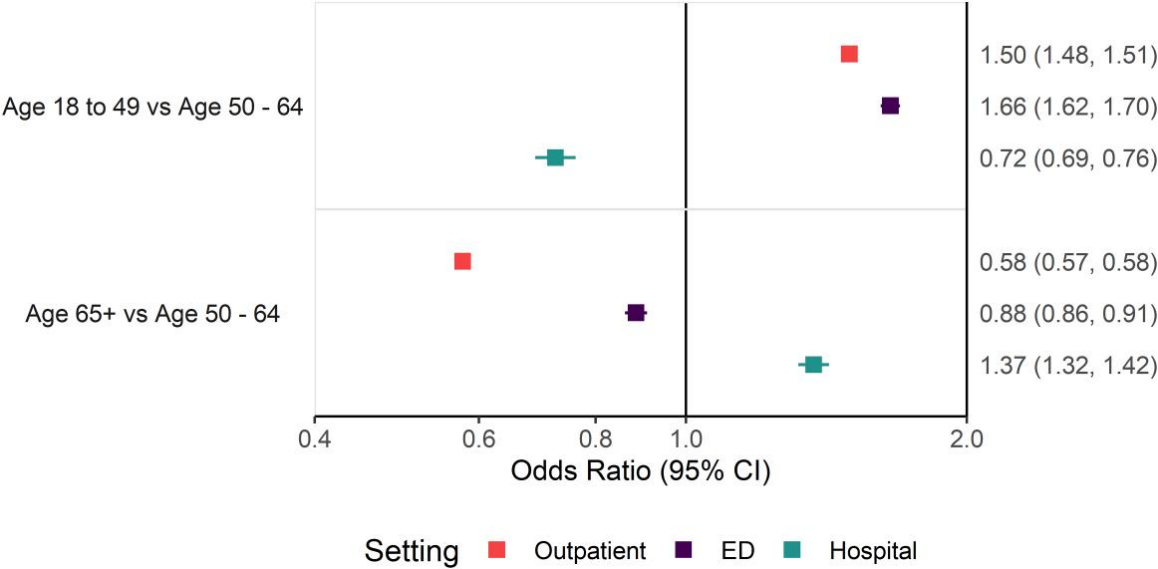
Age as a risk factor for influenza-related outpatient visits, emergency department (ED) visits, and hospitalizations.

As in the prior section, all results are for the overall population unless otherwise stated. Figure 3 shows that when compared with individuals 50 to 64 years old, those aged 18 to 49 years had higher odds of outpatient visits (OR, 1.50 [95% CI, 1.48–1.51]) and ED visits (1.66 [1.62–1.70]) but lower odds of hospitalization (0.72 [0.69–0.76]). When compared with those 50 to 64 years old, those aged ≥65 years had higher odds of hospitalizations (OR, 1.37 [95% CI, 1.32–1.42]) and lower odds of outpatient visits (0.58 [0.57–0.58]) and ED visits (0.88 [0.86–0.91]).

Relative to males, females had increased odds of influenza-related outpatient visits (OR, 1.10 [95% CI, 1.09–1.11]) and ED visits (1.24 [1.21–1.26]) but not hospitalizations (1.03 [1.00–1.07]; Supplementary Figure 2). Hispanic individuals had higher odds of having an influenza-related outpatient visit (OR, 1.12 [95% CI, 1.11–1.14]), ED visit (1.50 [1.46–1.55]), or hospitalization (1.15 [1.09–1.22]) when compared with non-Hispanic individuals (Supplementary Figure 3). Several significant differences in the odds of IRME were observed among racial groups (Supplementary Figure 4), with the largest difference observed between the Black and White groups. Specifically, patients who were Black had lower odds of an outpatient visit (OR, 0.70 [95% CI, 0.68–0.71]) but higher odds of an ED visit (1.68 [1.63–1.72]) or hospitalization (1.18 [1.12–1.24]) as compared with patients who were White.

All models adjusted for geographic region to account for potential geographic differences in health status (Supplementary Figure 4). Geographic differences in odds of IRME likely represent differences in health care availability and health care–seeking behavior rather than true differences in the risk of having an influenza infection.

### Clinical Risk Factors for IRME

Use of health care services in a specific setting during the off-season period was associated with higher odds of using services in that setting for influenza care when compared with not receiving care in that setting (Supplementary Figure 5). For example, those who had an outpatient visit in the off-season had higher odds of an influenza-related outpatient visit than those who did not have an outpatient visit during the off-season (OR, 1.42 [95% CI, 1.41–1.44]), whereas patients who had a hospitalization during the off-season had lower odds of an influenza-related outpatient visit than those who did not have a hospitalization during the off-season (0.87 [0.85–0.89]). In contrast, adults who had a hospitalization during the off-season period had increased odds of having an influenza-related hospitalization vs those who did not have a hospitalization during the off-season (OR, 1.96 [95% CI, 1.88–2.05]). In addition, for those who had a hospitalization in the off-season, the odds of an influenza-related hospitalization decreased with age (18–49 years: OR, 4.03 [95% CI, 3.64–4.45]; 50–64 years: 2.25 [2.09–2.44]; ≥65 years: 1.34 [1.26–1.44]). The higher likelihood of returning to the same clinical setting may be due to a combination of health care–seeking behavior/access and health status.

A 1-unit increase in Charlson Comorbidity Index was associated with decreased odds of having an influenza-related outpatient visit (OR, 0.96 [95% CI, 0.95–0.96]) and increased odds of an influenza-related ED visit (1.04 [1.03–1.05]) or hospitalization (1.11 [1.10–1.12]; Supplementary Figure 6). In the overall population, metabolic disorders and type 2 diabetes were associated with decreased odds of IRME, while systemic lupus erythematosus and rheumatoid arthritis were associated with increased odds of IRME. However, the trends for diabetes and systemic lupus erythematosus were not consistent and/or significant in all age subgroups.

A baseline hospitalization for ischemic stroke was associated with decreased odds of any type of IRME, whereas a baseline hospitalization for heart failure or a diagnosis of hypertension was associated with higher odds of an influenza-related ED visit or hospitalization (Supplementary Figure 7). Baseline hospitalizations for myocardial infarction or transient ischemic attacks were not associated with increased or decreased odds of IRME. No consistent age-related trends were observed among measures of cardiac health, although 95% CIs were wide due to the low incidence of cardiac-related baseline hospitalizations.

According to CDC standard cutoffs for BMI, patients who were underweight had higher odds of having an influenza-related ED visit (OR, 1.24 [95% CI, 1.13–1.35]) or hospitalization (1.50 [1.31–1.71]) when compared with those with a BMI in the healthy weight range (Supplementary Figure 8). By contrast, when healthy weight BMI was compared with overweight or obese, elevated BMI was associated with decreased odds of influenza-related hospitalization (overweight: OR, 0.86 [95% CI, 0.81–0.91]; obese: 0.88 [0.83–0.93]). Trends were generally consistent across age groups, though not always statistically significant.

When compared with never smokers, current smokers had decreased odds of an influenza-related outpatient visit but increased odds of an influenza-related ED visit (OR, 1.24 [95% CI, 1.17–1.31]) or hospitalization (1.21 [1.10–1.34]; Supplementary Figure 9). Effect sizes were smaller but followed the same trends when former smokers were compared with those who never smoked. Individuals with unknown smoking status had marginally higher odds of an IRME as compared with those who had never smoked. There were no clear trends for age, smoking status, and odds of IRME.

## DISCUSSION

In this analysis of 5 consecutive influenza seasons, adults with CDC-defined clinical risk factors were more likely to have an IRME than those with no risk factors across all age groups. The likelihood of having an IRME rose with the cumulative number of risk factors for all 3 outcomes (influenza-related outpatient visits, ED visits, and hospitalizations) and was significant even among patients with only 1 risk factor. The effect of additional risk factors on influenza-related ED visits and hospitalizations was observed in all age groups, though most pronounced among those aged 50 to 64 years. Other notable trends included that, relative to 50- to 64-year-olds, younger adults (18–49 years) had higher odds of influenza-related outpatient and ED visits, while older ones (≥65 years) had higher odds of influenza-related hospitalizations. Trends among clinical and nonclinical risk factors were of smaller magnitude or less consistent across age groups than for the cumulative number of risk factors, indicating that the number of CDC-defined clinical risk factors appears to be a more significant driver of IRME than age.

It has been well established that older adults and those with select comorbidities are at higher risk of severe influenza [4–6]. For example, in an analysis of influenza-related hospitalizations in England and Wales, older age and clinical risk (≥1 high-risk comorbidity) were associated with a high incidence of hospitalization and death [4]. There is less known about how the risk of medically attended influenza is influenced by multimorbidity. An estimated >25% of US adults and >50% of those aged >65 years have at least 2 chronic conditions [15], and the prevalence of multimorbidity has been rising over time [16, 17]. Although age is a strong predictor of multimorbidity, 23.7% of adults aged 30 to 64 years reported at least 2 chronic conditions in the National Health Interview Survey [18], and roughly 20% of those aged 18 to 49 years in this study had at least 2 risk factors.

Influenza vaccination is the best solution to prevent influenza and associated complications. While the decision to receive an influenza vaccine or not remains an individual's decision, being able to identify those at increased risk of influenza can help guide individual- and program-level decision making about influenza vaccination. It can also help direct resources for informational or outreach programs intended to improve influenza vaccination by focusing on those most at risk. The additive impact of multimorbidity on the likelihood of having influenza demonstrated in this study improves the ability to identify patients most at risk and provides a simple measure that may resonate with health care providers and their patients.

### Limitations and Strengths

One limitation of this study was that the claims data had a start date of 1 January 2015, resulting in a smaller sample size in the 2015–2016 season and incomplete baseline data for the 2015–2016 cohort. As a consequence, we may be underestimating the number of high-risk conditions among adults in the 2015–2016 season; however, the potential for bias has been reduced by the use of a fixed effect meta-analysis to estimate the effect size.

Another limitation is that certain CDC-identified risk factors for complications of influenza infection were not available or have low completeness in the data source. These include people living in a nursing home or other long-term care facility and people from certain racial or ethnic minority backgrounds. In addition, we used a BMI ≥30 as the highest category; however, the CDC identifies class III obesity (BMI ≥40) as a risk factor for influenza. This may have reduced our ability to detect the effect of obesity on the odds of medically attended influenza.

In the analysis of the impact of the number of CDC-defined risk factors on odds of different IRMEs, we adjusted for the presence of specific risk factors, which can help adjust for imbalances in the conditions that contributed to that count. Yet, it is possible that specific combinations of risk factors had a greater combined impact than what would be assumed based on the effect of the individual risk factors, which would not be completely adjusted for.

When the analyses were stratified by age group, any of the comparisons made were among 1 age group. For example, when 0 vs 1 risk factor was compared in an age group, it was only among people in that age stratum. There are likely health differences among age groups, even among people with a certain number of risk factors, which limits our ability to directly compare the impact of any given risk factor among age groups.

A final limitation is that this study is measuring influenza health care encounters and not influenza infections, and health care–seeking behavior may vary by demographic and clinical characteristics. For instance, prior studies found that smokers were less likely to use primary care services [19] and patients with comorbidities were more likely to seek care for influenza-like illnesses [20]. This study also found that current/former smokers were less likely to have an outpatient visit for influenza but increased likelihood of ED visits or hospitalization. The lower likelihood of having an influenza-related outpatient visit likely represents current/former smokers being less likely to seek care in general and not a reduction in their likelihood of having a symptomatic influenza infection.

The strengths of our study include the use of data from a large, geographically distributed population. In addition, we took steps to increase data completeness by using linked EHR and claims data and restricting to individuals with known age, gender, and geography. Finally, the analysis was repeated across 5 seasons, and the effect size was estimated via a fixed effect meta-analysis.

## CONCLUSIONS

In the current study, we observed a clear trend toward higher odds of IRME with an increasing number of high-risk comorbidities. These results show that in any age group, a simple measure such as the number of CDC influenza risk factors can be highly informative of an individual's potential for severe influenza outcomes and may be informative for individual- and program-level decisions on preventive measures, including influenza vaccination.

## Supplementary Material

ofae203_Supplementary_Data

## Supplementary Data

Supplementary materials are available at *Open Forum Infectious Diseases* online. Consisting of data provided by the authors to benefit the reader, the posted materials are not copyedited and are the sole responsibility of the authors, so questions or comments should be addressed to the corresponding author.

## Notes

***Acknowledgments*.** We thank Mahrukh Imran (CSL Seqirus) for her contribution to the manuscript. Jessamine Winer-Jones, an employee of Veradigm, provided medical writing support for this manuscript, which was funded by CSL Seqirus.

***Author contributions*.** I. M. and M. D. M. H. contributed to study conceptualization. All authors contributed to study design, analysis, and interpretation. K. C. and A. N. B. contributed to data collection and data processing. I. M. led development of the first draft of the manuscript. All authors revised the manuscript critically, made substantive intellectual contributions to the development of this manuscript, and approved the final version.

***Data availability*.** The data that support the findings of this study were used under license from Veradigm and Komodo Health. Due to data use agreements and their proprietary nature, restrictions apply regarding the availability of the data. Further information is available from the corresponding author.

***Patient consent statement*.** The linked EHR and claims data set used in this study has been certified as statistically deidentified through a formal determination by a qualified expert as defined in §164.514(b)(1) of the privacy rule of the Health Insurance Portability and Accountability Act of 1996. Because this study used only deidentified patient records, it is no longer subject to the privacy rule and is therefore exempt from institutional review board approval and for obtaining informed consent according to US law.

***Financial support*.** This work was supported by CSL Seqirus.
