# Supplementary material for: Number of Influenza Risk Factors Informs an Adult's Increased Potential of Severe Influenza Outcomes: A Multiseason Cohort Study From 2015 to 2020
Source: Open Forum Infect Dis. 2024 Apr 12;11(5):ofae203. doi: 10.1093/ofid/ofae203 (PMC11083624; doi:10.1093/ofid/ofae203)
Supplement: ofae203_Supplementary_Data [file ofae203_supplementary_data.docx]

Supplementary material for: The Number of Influenza Risk Factors Informs an Adult's Increased Potential of Severe Influenza Outcomes: A Multi-Season Cohort Study from 2015 to 2020.

**Authors**: Ian McGovern^1^, Katherine Cappell^2^, Alina N Bogdanov^2^, Mendel DM Haag^3^

**Affiliation:** ^1^CSL Seqirus, Waltham, MA, USA; ^2^Veradigm, Chicago, IL, USA; ^3^CSL Seqirus, Amsterdam, The Netherlands

Supplementary Figure 1. Study Time Periods

12 Month Baseline Period

Start Week 40

Year 0

Start Week 40

Year -1

End Week 20

Year 1

Off-season period

Weeks 21-39 of Year 0

Influenza Season

Supplementary Figure 2. Sex as a Risk Factor for Influenza-Related Outpatient Visits, Emergency Room (ER) Visits, and Hospitalizations
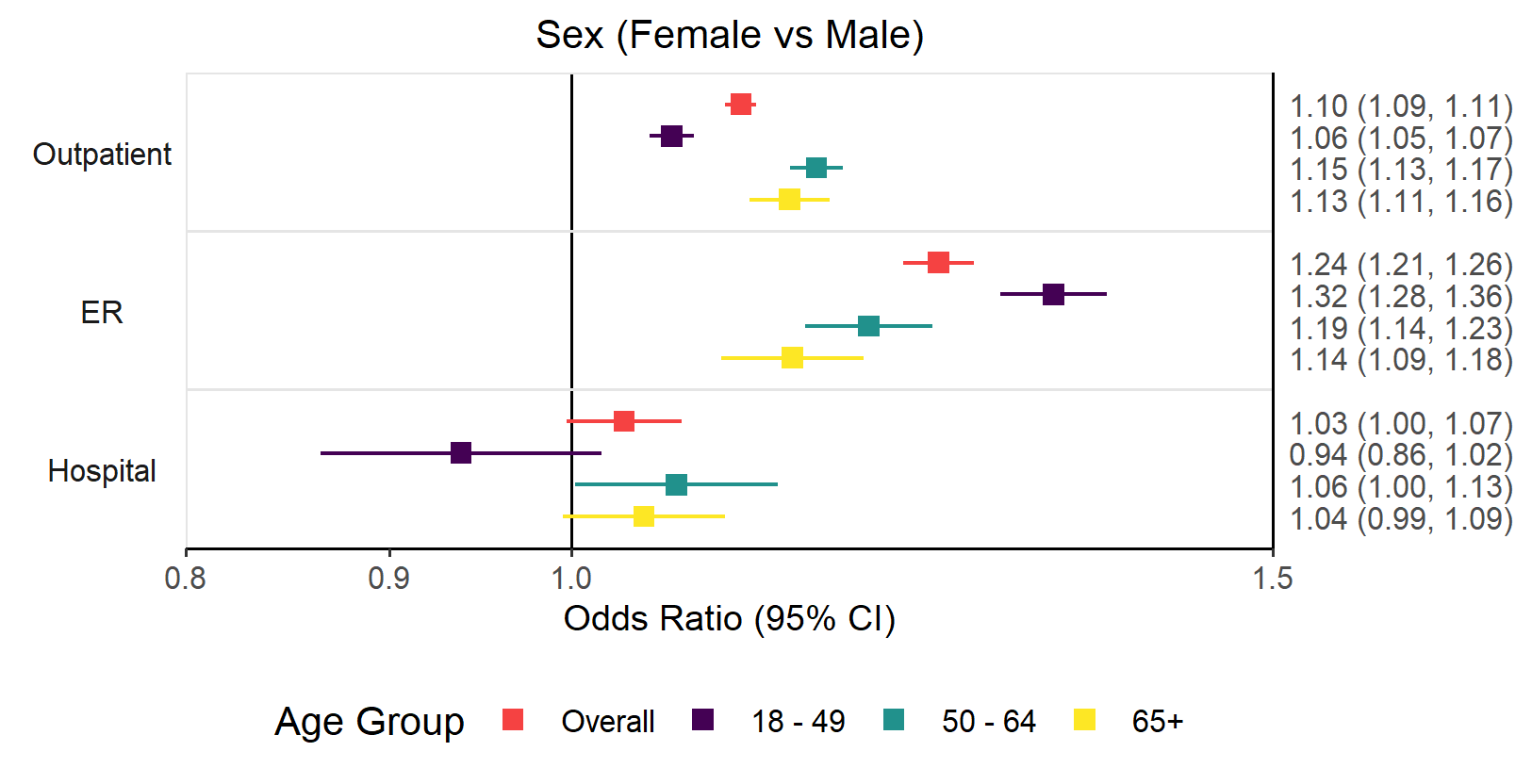


Supplementary Figure 3. Race and Ethnicity as a Risk Factor for Influenza-Related Outpatient Visits, Emergency Room Visits, and Hospitalizations


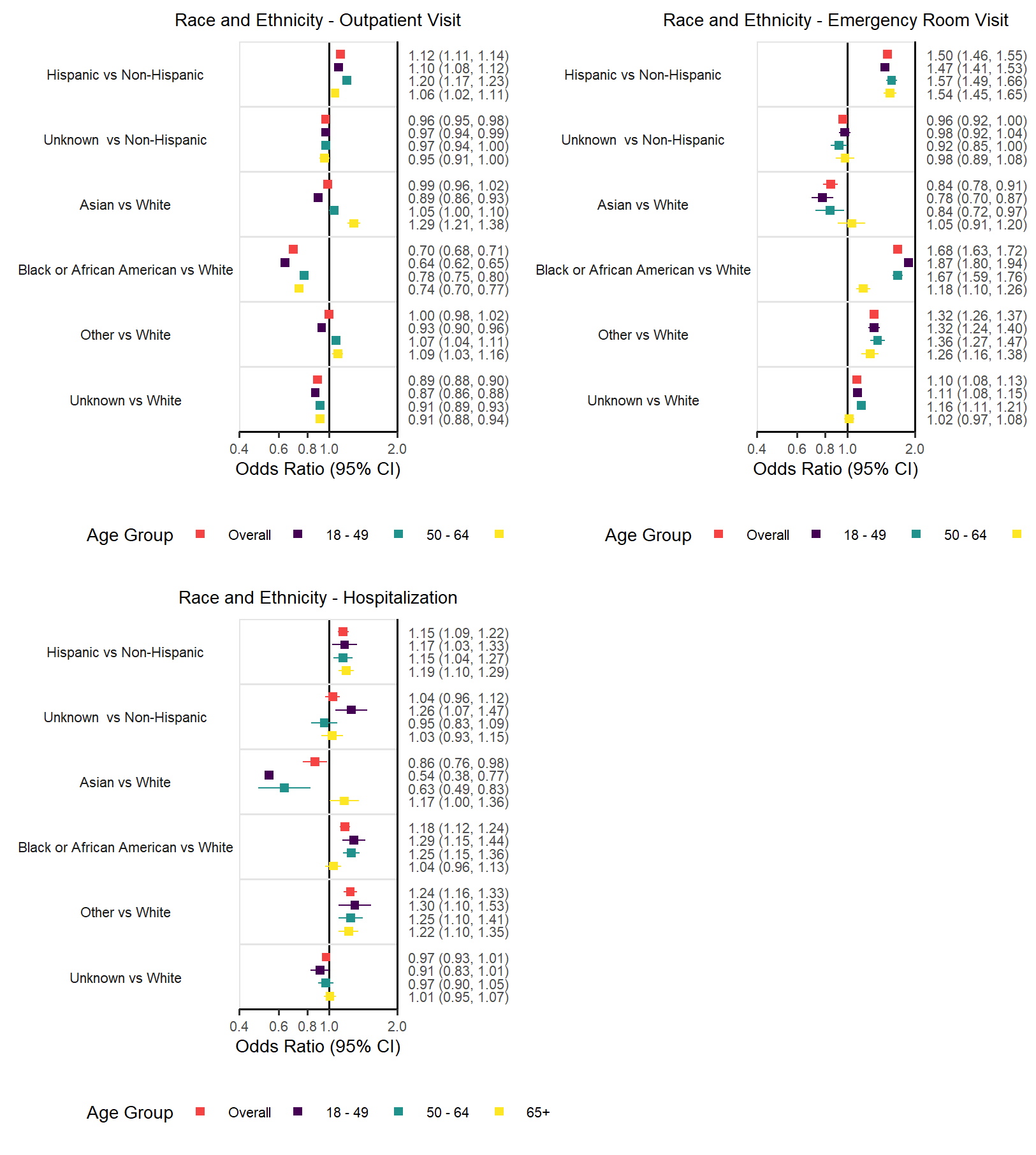


Supplementary Figure 4. Geographic Region as a Risk Factor for Influenza-Related Outpatient Visits, Emergency Room Visits, and Hospitalizations


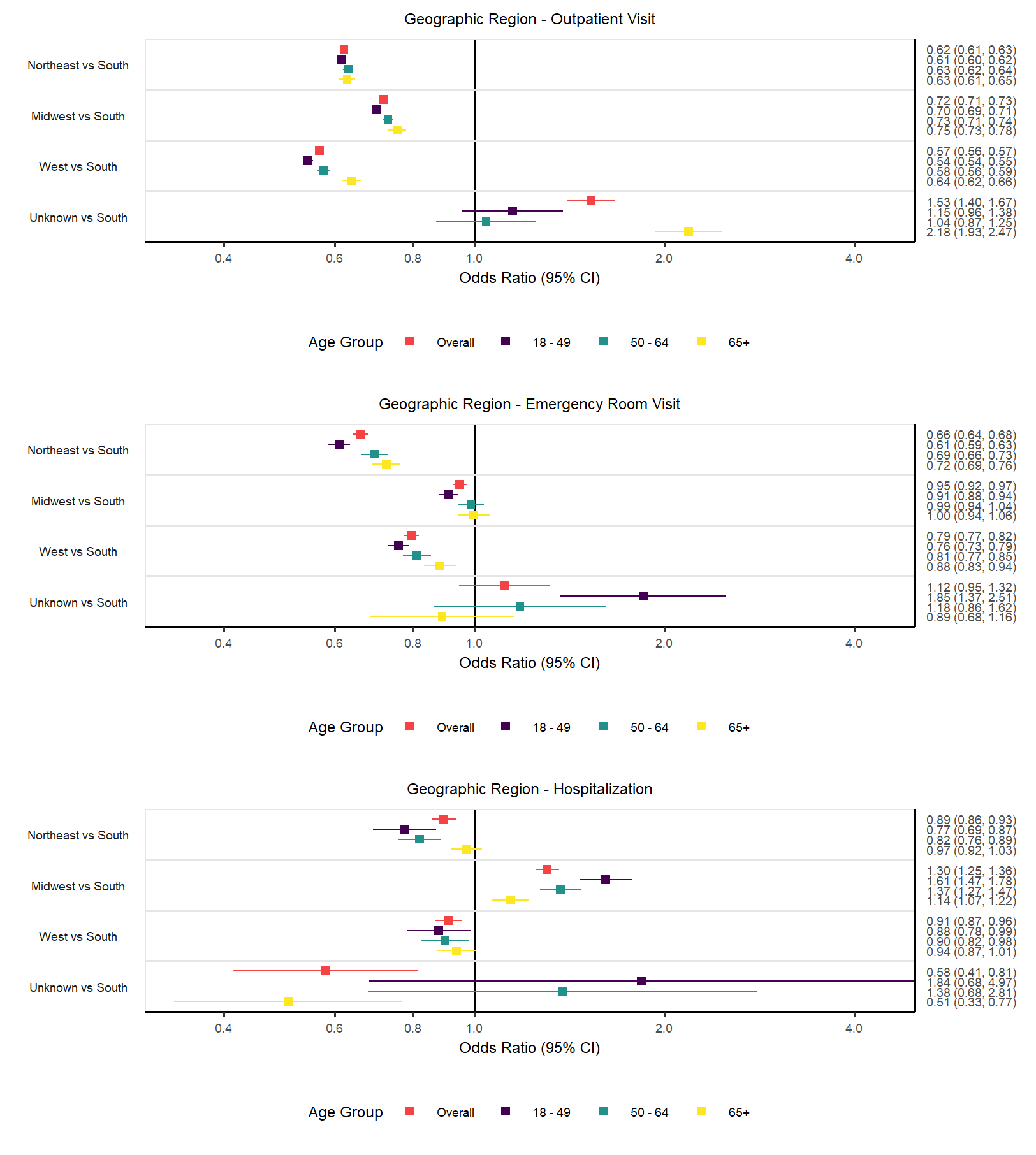


Supplementary Figure 5. Off-season Healthcare Resource Use as a Risk Factor for Influenza-Related Outpatient Visits, Emergency Room Visits, and Hospitalizations


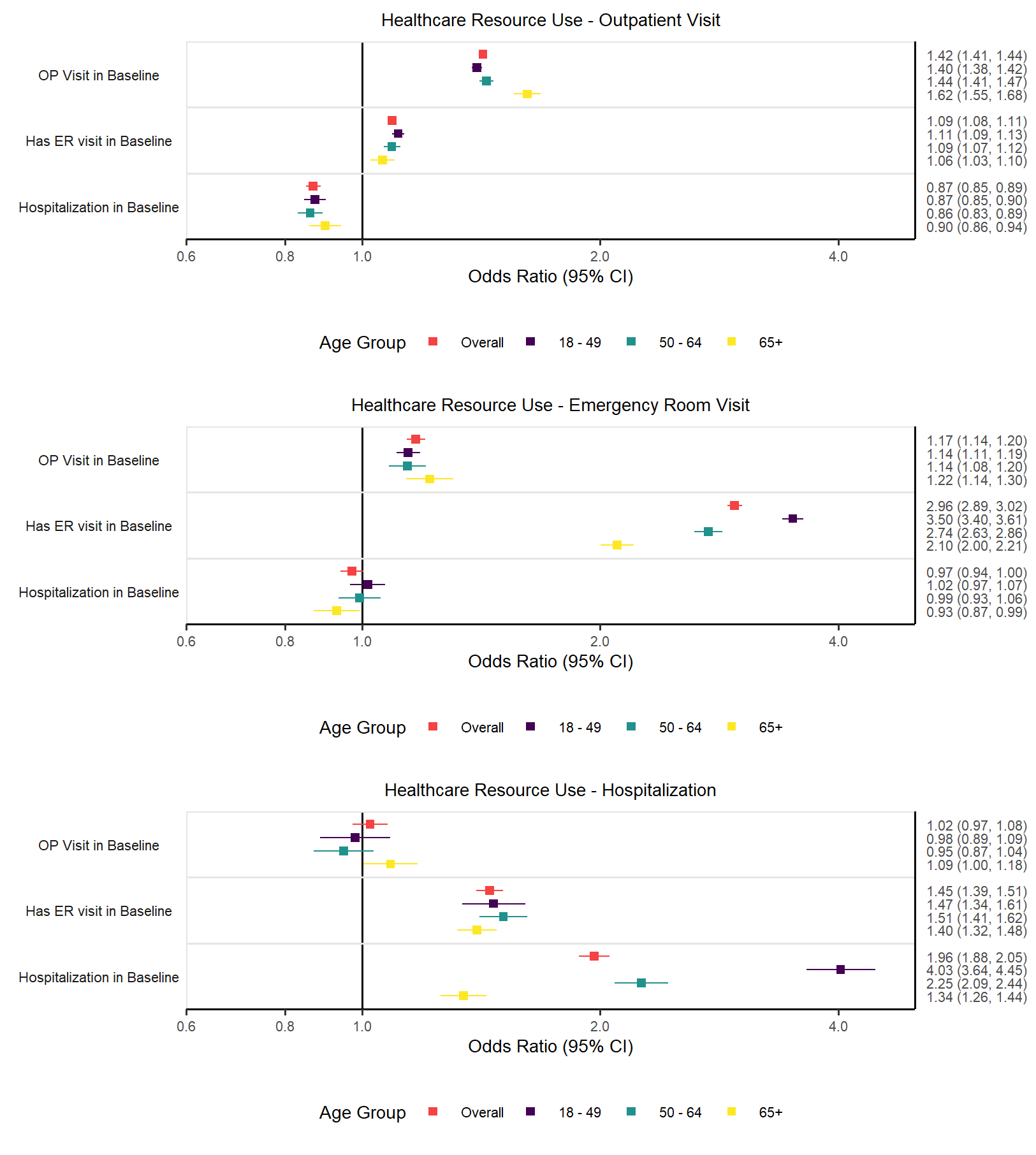


Supplementary Figure 6. Charlson Comorbidity Index (CCI) and Other Individual Comorbidities as a Risk Factor for Influenza-Related Outpatient Visits, Emergency Room Visits, and Hospitalizations. CCI, Charlson comorbidity index


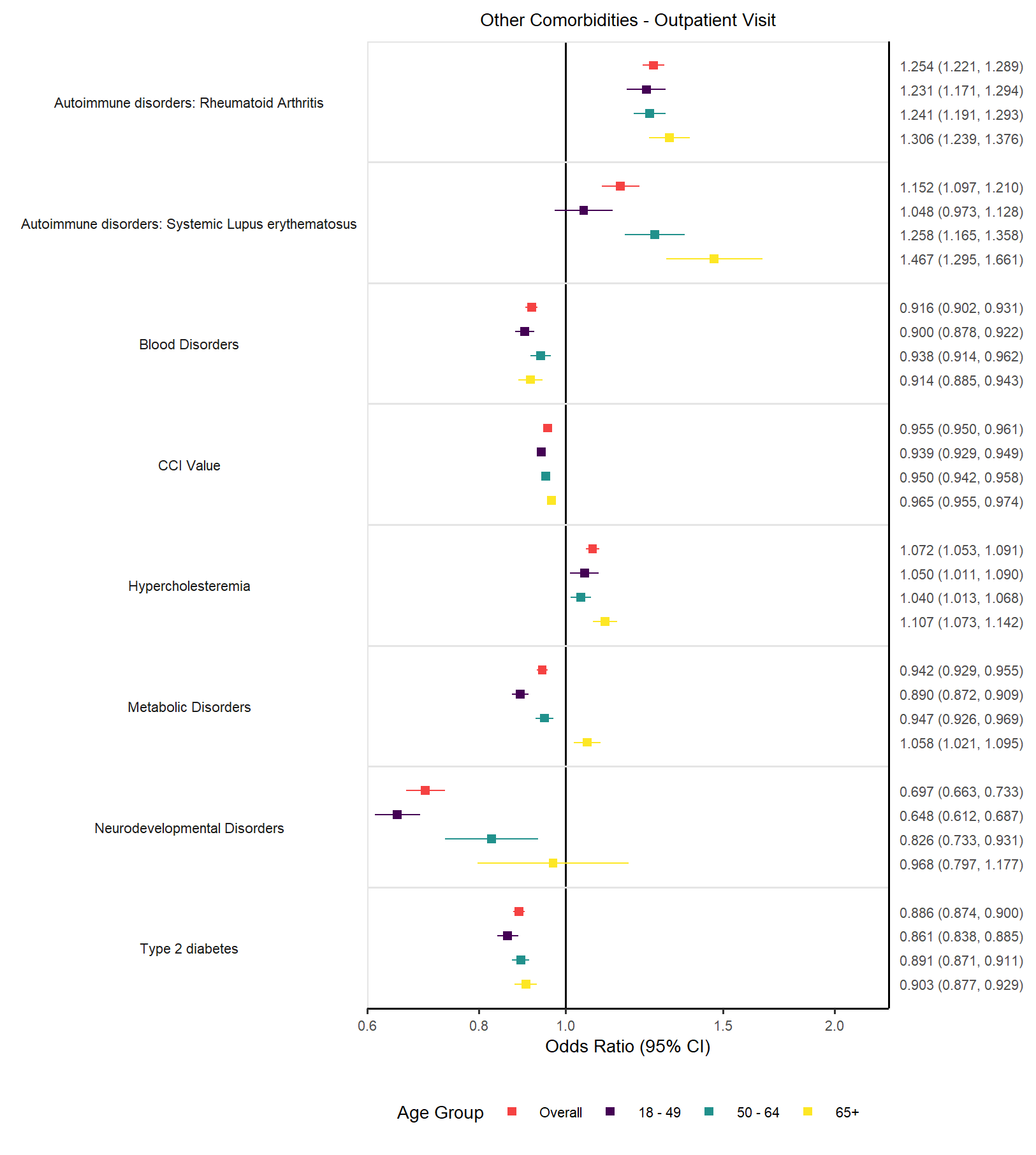


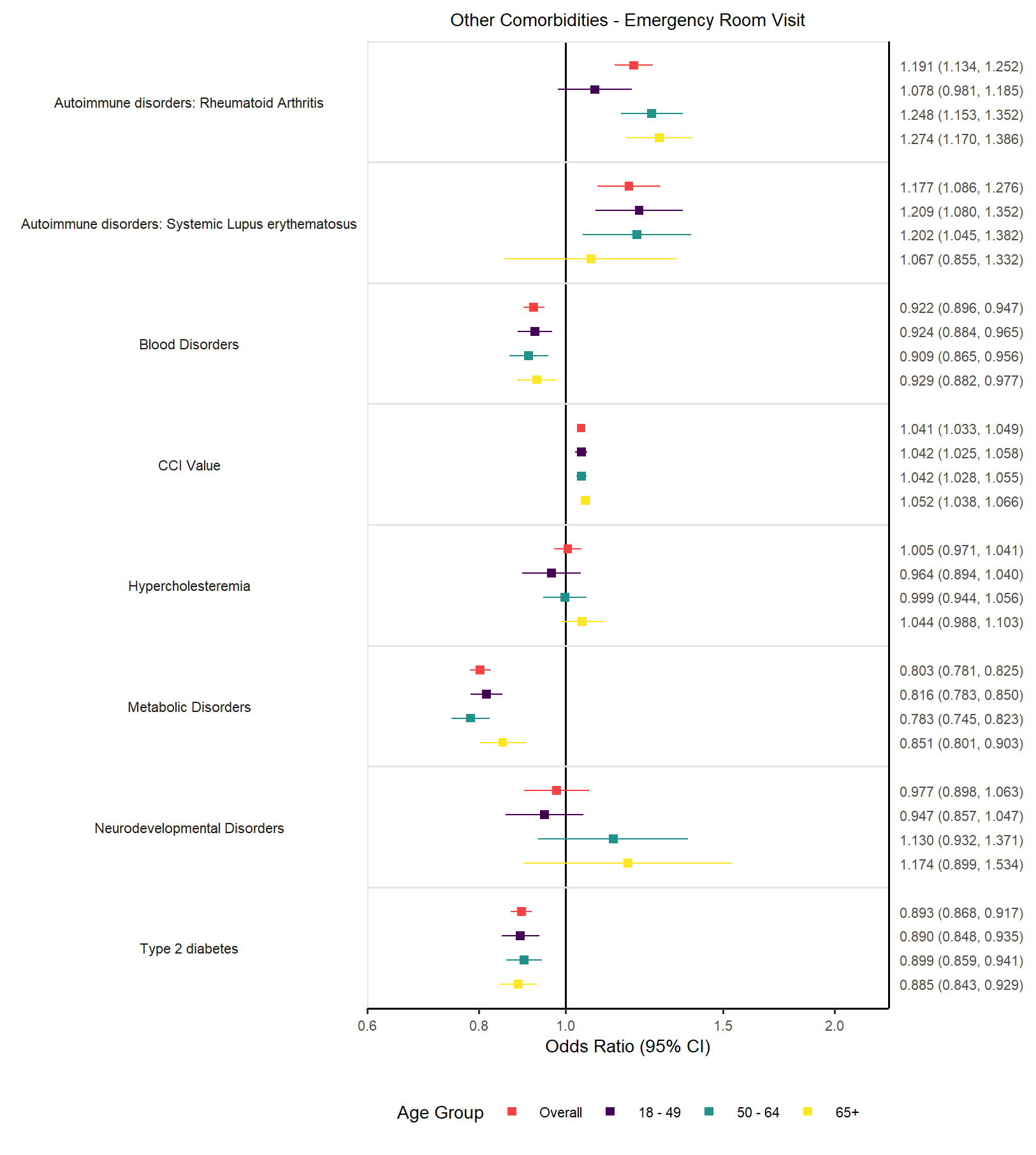


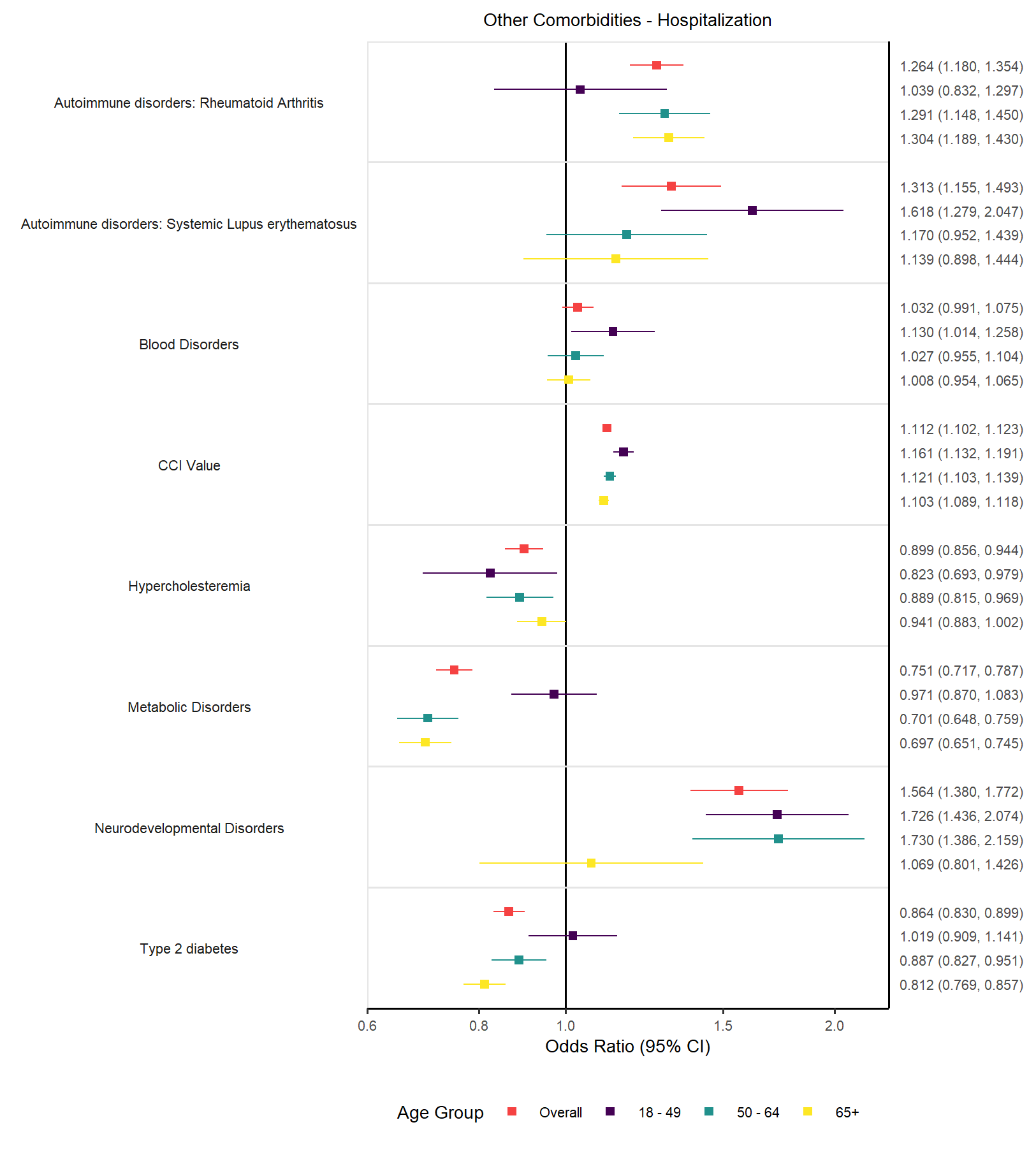


Supplementary Figure 7. Cardiovascular Hospitalizations or Diagnosis as a Risk Factor for Influenza-Related Outpatient Visits, Emergency Room Visits, and Hospitalizations. MI, myocardial infarction; TIA, transient ischemic attacks


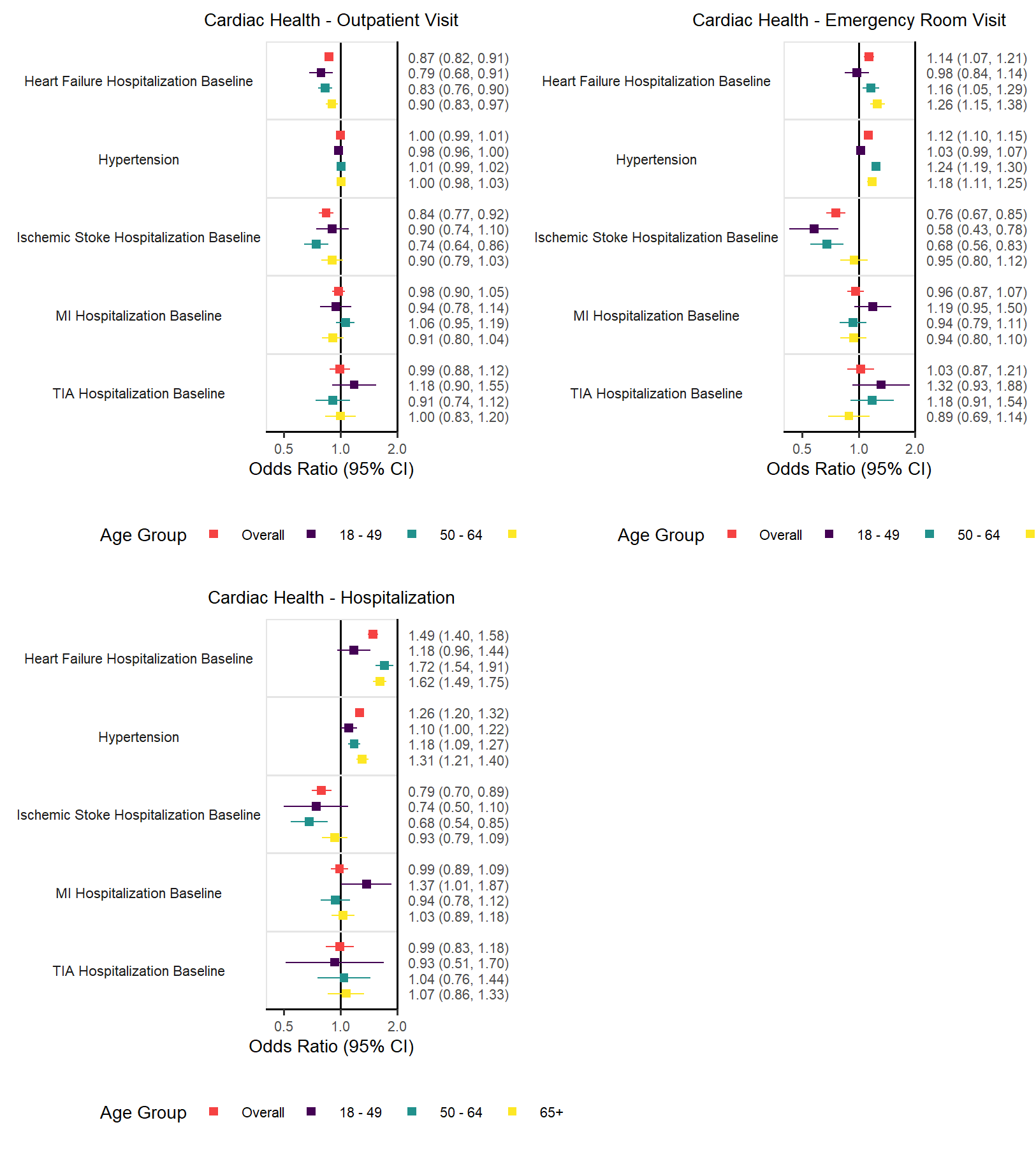


Supplementary Figure 8. Body Mass Index (BMI) as a Risk Factor for Influenza-Related Outpatient Visits, Emergency Room Visits, and Hospitalizations. BMI, Body Mass Index


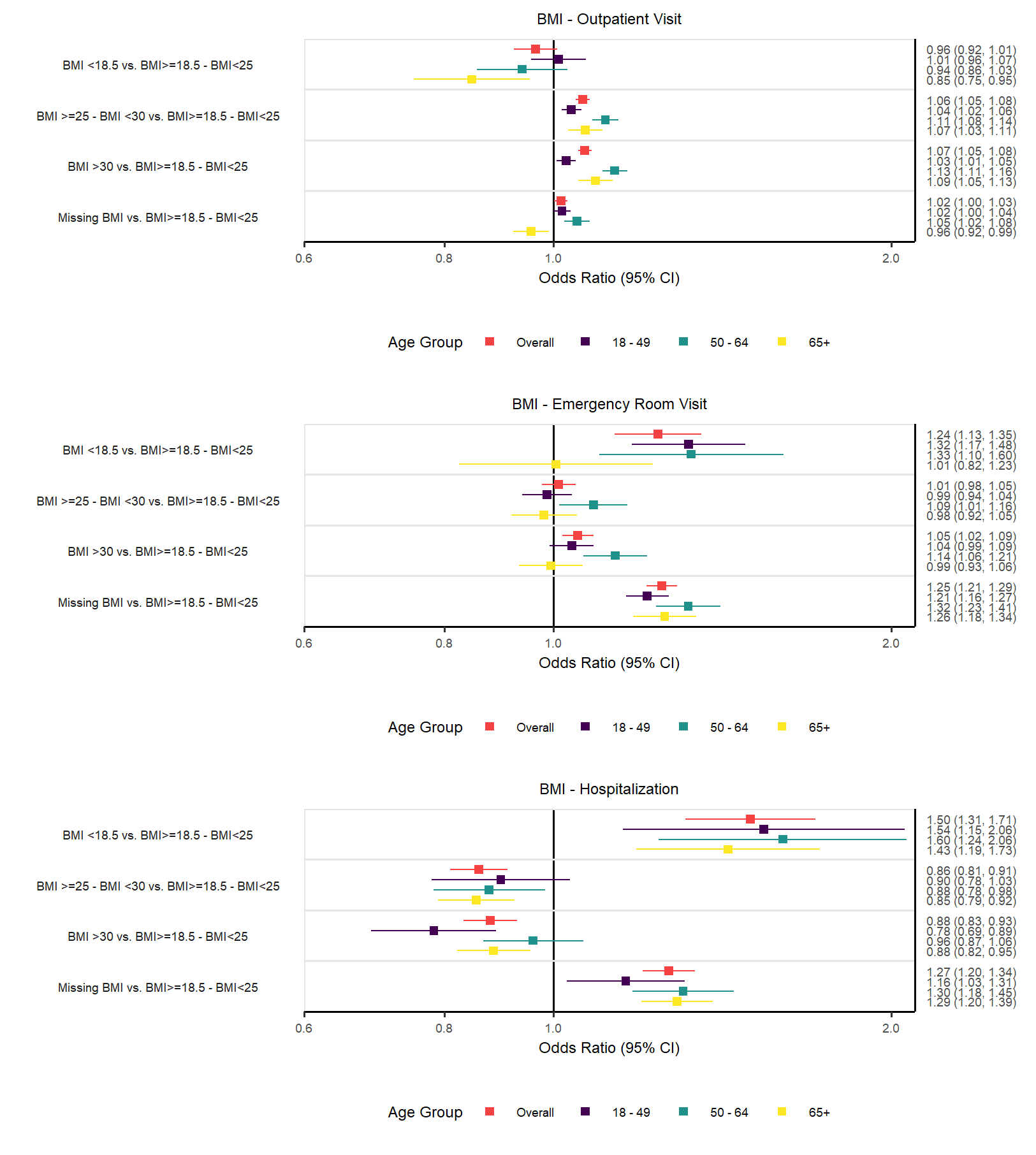


Supplementary Figure 9. Smoking History as a Risk Factor for Influenza-Related Outpatient Visits, Emergency Room Visits, and Hospitalizations


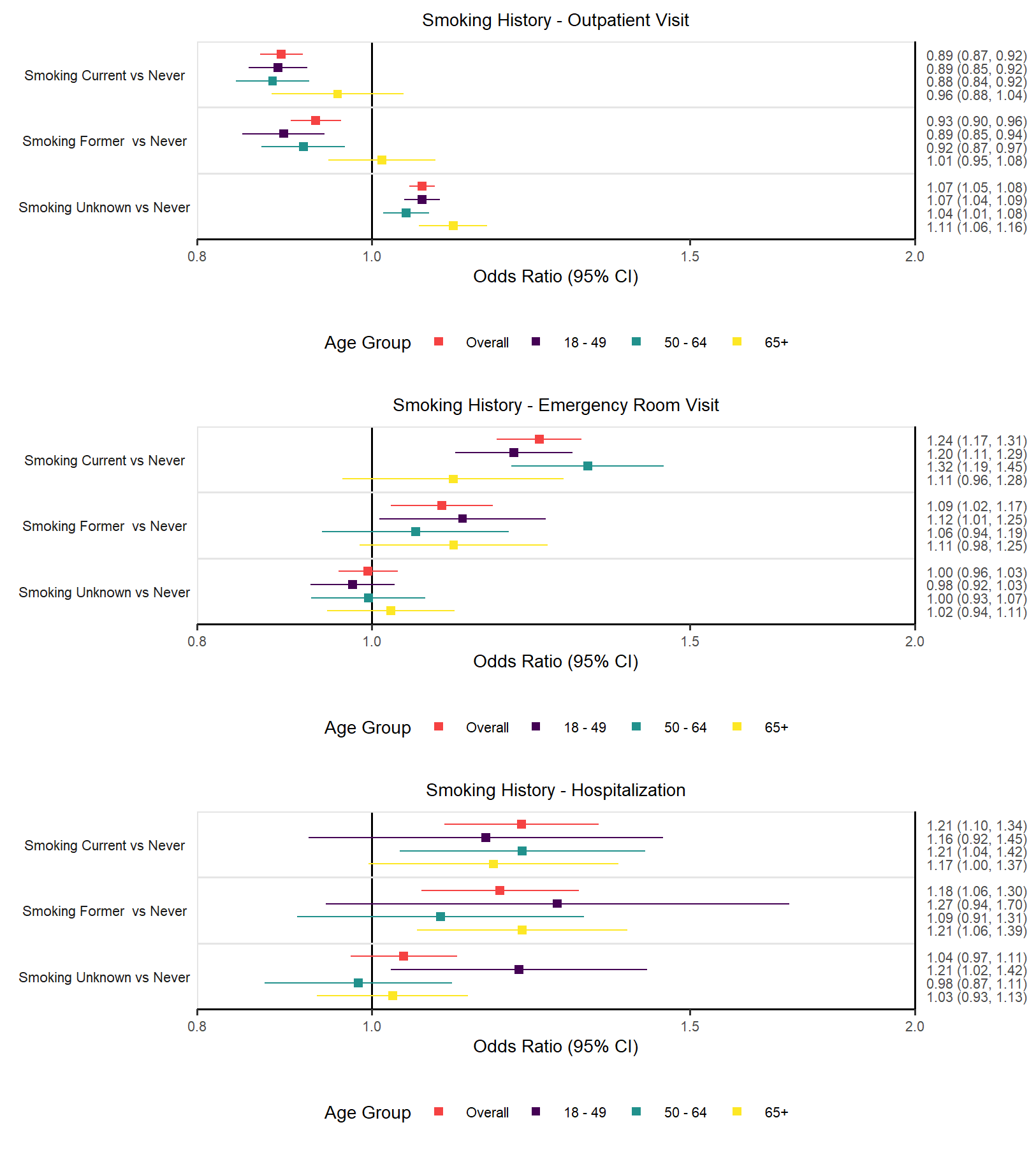


Supplementary Table 1. Study Measures

| Influenza risk factors | - Asthma - Neurologic and neurodevelopmental conditions - Blood disorders (e.g., sickle cell disease) - Chronic lung disease (e.g., chronic obstructive pulmonary disease, cystic fibrosis) - Endocrine disorders (e.g., diabetes mellitus) - Heart disease (e.g., congenital heart disease, congestive heart failure, coronary artery disease) - Kidney diseases - Liver disorders - Metabolic disorders (e.g., inherited metabolic disorders, mitochondrial disorders) - Obesity (body mass index of 40 or higher) - Weakened immune system due to disease (e.g., HIV or AIDS, leukemia) or medications (e.g., chemotherapy or radiation treatment for cancer, chronic corticosteroids) - Stroke |
| --- | --- |
| Demographic characteristics | - Age at Start of Season - Sex (Female, Male) - Race (Black or African American, White, Other, Not Reported) - Ethnicity (Hispanic, Non-Hispanic, Not Reported) - Geographic region (Northeast, Midwest, South, West, Other) |
| Clinical characteristics | - Influenza Vaccination Status - All-cause healthcare resource utilization in the off-season period - Outpatient visits - Emergency department visits - Hospitalizations - Health status, defined by: - Charlson co-morbidity index and individual contributing conditions - Specific high-risk medical conditions for influenza complication   - Metabolic disorders   - Blood disorders   - Neurologic and neurodevelopmental conditions - Baseline cardiovascular risk:   - Cardiovascular inpatient events: myocardial infarction, ischemic stroke, heart failure, and transient ischemic attack.   - Cardiovascular-related diagnoses: hypercholesteremia, hypertension, type 2 diabetes   - Autoimmune disorders: systemic lupus erythematosus and rheumatoid arthritis   - BMI and smoking status |

Supplementary Table 2: Study Subject Selection by Season

|  | **2015–2016** | **2016–2017** | **2017–2018** | **2018–2019** | **2019–2020** |
| --- | --- | --- | --- | --- | --- |
| 1) Individual had a transcript record in the Veradigm EMR during the influenza season | 34,545,275 | 33,699,265 | 32,825,827 | 29,136,217 | 23,999,757 |
| 2) Individual had a transcript record in the Veradigm EMR at least 1 year prior to start of influenza season | 19,601,796 (56.7%) | 21,736,823 (64.5%) | 22,152,225 (67.5%) | 20,637,829 (70.8%) | 18,033,597 (75.1%) |
| 3) Individual was continuously enrolled in the claims data from at least 1 year prior to the start of the influenza season to 120 days after the end of the influenza season | 1,354,241 (6.9%) | 4,105,800 (18.9%) | 4,517,236 (20.4%) | 4,805,530 (23.3%) | 4,268,586 (23.7%) |
| 4) Individual did not have an influenza-related medical encounter in weeks 21–39 prior to the start of the influenza season | 1,351,834 (99.8%) | 4,099,890 (99.9%) | 4,509,829 (99.8%) | 4,796,370 (99.8%) | 4,257,243 (99.7%) |
| 5) Individual was at least 18 years old at start of influenza season | 1,118,466 (82.7%) | 3,410,121 (83.2%) | 3,768,633 (83.6%) | 4,130,019 (86.1%) | 3,700,784 (86.9%) |
| 6) Individual did not have missing gender and geographic region data | 887,260 (79.3%) | 2,789,372 (81.8%) | 3,202,455 (85.0%) | 3,628,168 (87.8%) | 3,310,936 (89.5%) |
| Patients aged 18–49 | 392,647 (44.3%) | 1,130,716 (40.5%) | 1,285,802 (40.2%) | 1,408,171 (38.8%) | 1,252,459 (37.8%) |
| Patients aged 50–64 | 342,439 (38.6%) | 1,003,938 (36.0%) | 1,129,708 (35.3%) | 1,249,587 (34.4%) | 1,108,895 (33.5%) |
| Patients aged ≥65 | 152,174 (17.2%) | 654,718 (23.5%) | 786,945 (24.6%) | 970,410 (26.7%) | 949,582 (28.7%) |

Supplementary Table 3. Baseline Characteristics of Individuals Included in (A) the 2015–2016 Season, (B) the 2016–2017 Season, (C) the 2017–2018 Season, (D) the 2018–2019 Season, and (E) the 2019–2020 Season.

| Supplementary Table 2A: 2015–2016 Season | | | | | |
| --- | --- | --- | --- | --- | --- |
|  | 18–49 Yrs | 50–64 Yrs | ≥65 Yrs | SMD 50–64 vs.  18–49 | SMD 50–64 vs. ≥65 |
|  | N = 392,647 | N = 342,439 | N = 152,174 |  |  |
| Age, mean (SD) | 35.6 (9.3) | 57.0 (4.1) | 72.3 (5.8) | **7.223** | **-5.751** |
| Age, median (IQR) | 37 (28 – 44) | 57 (53 – 61) | 71 (67 – 77) |  |  |
| Sex, N (%) |  |  |  |  |  |
| Female | 251,269 (64.0%) | 200,664 (58.6%) | 86,988 (57.2%) | **-0.111** | 0.029 |
| Male | 141,378 (36.0%) | 141,775 (41.4%) | 65,186 (42.8%) | **0.111** | -0.029 |
| Race, N (%) |  |  |  |  |  |
| Asian | 10,883 (2.8%) | 7,418 (2.2%) | 3,789 (2.5%) | -0.039 | -0.021 |
| Black or African American | 32,235 (8.2%) | 26,024 (7.6%) | 11,046 (7.3%) | -0.023 | 0.013 |
| Other | 14,042 (3.6%) | 10,874 (3.2%) | 5,924 (3.9%) | -0.022 | -0.039 |
| Unknown | 81,602 (20.8%) | 56,450 (16.5%) | 22,291 (14.6%) | **-0.111** | 0.051 |
| White | 253,885 (64.7%) | 241,673 (70.6%) | 109,124 (71.7%) | **0.127** | -0.025 |
| Ethnicity, N (%) |  |  |  |  |  |
| Hispanic | 33,367 (8.5%) | 19,956 (5.8%) | 10,525 (6.9%) | **-0.104** | -0.045 |
| Non-Hispanic | 324,890 (82.7%) | 294,766 (86.1%) | 130,965 (86.1%) | 0.092 | 0.000 |
| Unknown/not reported | 34,390 (8.8%) | 27,717 (8.1%) | 10,684 (7.0%) | -0.024 | 0.041 |
| Geographic region, N (%) |  |  |  |  |  |
| Northeast | 90,810 (23.1%) | 85,305 (24.9%) | 46,589 (30.6%) | 0.042 | -0.128 |
| Midwest | 91,374 (23.3%) | 79,049 (23.1%) | 34,638 (22.8%) | -0.004 | 0.008 |
| South | 146,816 (37.4%) | 125,720 (36.7%) | 50,400 (33.1%) | -0.014 | 0.075 |
| West | 63,644 (16.2%) | 52,351 (15.3%) | 20,492 (13.5%) | -0.025 | 0.052 |
| Other/unknown | 3 (0.0%) | 14 (0.0%) | 55 (0.0%) | 0.007 | -0.023 |
| Influenza vaccination status, N (%) |  |  |  |  |  |
| No | 326,651 (83.2%) | 253,508 (74.0%) | 103,018 (67.7%) | **-0.225** | **0.140** |
| Yes | 65,996 (16.8%) | 88,931 (26.0%) | 49,156 (32.3%) | **0.225** | **-0.140** |
| Healthcare resource utilization in off-season period |  |  |  |  |  |
| Patients with OP visit, N (%) | 266,703 (67.9%) | 261,764 (76.4%) | 128,321 (84.3%) | **0.191** | **-0.200** |
| Number of all-cause OP visits, mean (SD) | 2.9 (3.4) | 3.2 (3.8) | 3.8 (4.4) | **0.136** | **-0.210** |
| Patients with ER visit, N (%) | 42,998 (11.0%) | 28,933 (8.4%) | 15,681 (10.3%) | -0.085 | -0.064 |
| Number of all-cause ER visits, mean (SD) | 1.9 (2.7) | 1.9 (3.0) | 2.0 (3.2) | -0.003 | -0.069 |
| Patients with hospitalizations, N (%) | 12,715 (3.2%) | 12,786 (3.7%) | 10,466 (6.9%) | 0.027 | **-0.141** |
| Number of all-cause hospitalizations, mean (SD) | 1.3 (1.0) | 1.4 (1.0) | 1.4 (1.1) | 0.057 | -0.045 |
| Baseline health status |  |  |  |  |  |
| High-risk patients, N (%) | 125,008 (31.8%) | 187,827 (54.8%) | 114,095 (75.0%) | **0.477** | -**0.431** |
| 1 High risk condition | 72,111 (18.4%) | 101,727 (29.7%) | 45,898 (30.2%) | **0.268** | -0.010 |
| 2 High risk conditions | 32,989 (8.4%) | 46,591 (13.6%) | 30,015 (19.7%) | **0.167** | **-0.165** |
| 3 High risk conditions | 12,617 (3.2%) | 22,066 (6.4%) | 18,510 (12.2%) | **0.151** | **-0.198** |
| =>4 High risk conditions | 7,291 (1.9%) | 17,443 (5.1%) | 19,672 (12.9%) | **0.177** | **-0.276** |
| CCI, mean (SD) | 0.2 (0.7) | 0.6 (1.2) | 1.3 (1.8) | **0.272** | **-0.475** |
| CCI conditions, N (%) |  |  |  |  |  |
| Any malignancy | 5,798 (1.5%) | 18,485 (5.4%) | 17,137 (11.3%) | **0.216** | **-0.213** |
| Congestive heart failure | 2,600 (0.7%) | 8,746 (2.6%) | 12,769 (8.4%) | **0.151** | **-0.259** |
| Cerebrovascular disease | 2,991 (0.8%) | 9,806 (2.9%) | 14,535 (9.6%) | **0.158** | **-0.280** |
| Chronic pulmonary disease | 32,034 (8.2%) | 39,538 (11.5%) | 26,211 (17.2%) | **0.114** | **-0.162** |
| Dementia | 249 (0.1%) | 856 (0.2%) | 4,910 (3.2%) | 0.047 | **-0.229** |
| Diabetes with chronic complication | 3,950 (1.0%) | 13,331 (3.9%) | 15,170 (10.0%) | **0.188** | **-0.241** |
| Diabetes without chronic complication | 3,155 (0.8%) | 7,227 (2.1%) | 6,513 (4.3%) | **0.109** | **-0.124** |
| Hemiplegia or paraplegia | 1,662 (0.4%) | 1,478 (0.4%) | 1,074 (0.7%) | 0.001 | -0.036 |
| Mild liver disease | 7,605 (1.9%) | 13,273 (3.9%) | 5,558 (3.7%) | **0.116** | 0.012 |
| Moderate to severe liver disease | 284 (0.1%) | 830 (0.2%) | 360 (0.2%) | 0.043 | 0.001 |
| Myocardial infarction | 1,111 (0.3%) | 4,962 (1.4%) | 5,563 (3.7%) | **0.126** | **-0.140** |
| Metastatic solid tumor | 499 (0.1%) | 1,579 (0.5%) | 1,096 (0.7%) | 0.062 | -0.034 |
| Peripheral vascular disease | 2,436 (0.6%) | 10,330 (3.0%) | 17,393 (11.4%) | **0.180** | **-0.329** |
| Peptic ulcer disease | 1,227 (0.3%) | 1,940 (0.6%) | 1,505 (1.0%) | 0.038 | -0.048 |
| Renal disease, mild to moderate | 3,584 (0.9%) | 10,506 (3.1%) | 17,050 (11.2%) | **0.155** | **-0.320** |
| Severe renal disease | 1,151 (0.3%) | 2,460 (0.7%) | 2,748 (1.8%) | 0.060 | -0.098 |
| Rheumatic disease | 5,234 (1.3%) | 9,492 (2.8%) | 5,931 (3.9%) | **0.102** | -0.063 |
| HIV infection, no AIDS | 1,409 (0.4%) | 1,470 (0.4%) | 220 (0.1%) | 0.011 | 0.053 |
| AIDS | 209 (0.1%) | 213 (0.1%) | 25 (0.0%) | 0.004 | 0.023 |
| Additional High Risk Comorbidities, N (%) |  |  |  |  |  |
| Metabolic disorders | 56,607 (14.4%) | 136,268 (39.8%) | 91,793 (60.3%) | **0.596** | **-0.419** |
| Blood disorders | 21,534 (5.5%) | 26,438 (7.7%) | 23,526 (15.5%) | 0.090 | **-0.244** |
| Neurologic and neurodevelopmental conditions | 2,992 (0.8%) | 653 (0.2%) | 234 (0.2%) | -0.083 | 0.009 |
| Baseline cardiovascular risk, N (%) |  |  |  |  |  |
| Myocardial infarction-related hospitalizations | 295 (0.1%) | 1,187 (0.3%) | 1,089 (0.7%) | 0.059 | -0.051 |
| Ischemic stroke-related hospitalizations | 252 (0.1%) | 733 (0.2%) | 947 (0.6%) | 0.040 | -0.063 |
| Heart failure-related hospitalizations | 533 (0.1%) | 1,874 (0.5%) | 2,999 (2.0%) | 0.071 | **-0.128** |
| Transient ischemic attack-related hospitalizations | 109 (0.0%) | 347 (0.1%) | 444 (0.3%) | 0.029 | -0.043 |
| Hypercholesteremia | 10,552 (2.7%) | 33,505 (9.8%) | 26,336 (17.3%) | **0.297** | **-0.221** |
| Hypertension | 52,065 (13.3%) | 132,066 (38.6%) | 95,223 (62.6%) | **0.603** | **-0.495** |
| Type 2 diabetes | 21,932 (5.6%) | 58,538 (17.1%) | 44,401 (29.2%) | **0.369** | **-0.290** |
| Systemic lupus erythematosus | 1,841 (0.5%) | 1,730 (0.5%) | 557 (0.4%) | 0.005 | 0.021 |
| Rheumatoid arthritis | 3,579 (0.9%) | 7,396 (2.2%) | 4,612 (3.0%) | **0.102** | -0.055 |
| Has BMI, N (%) | 250,055 (63.7%) | 240,708 (70.3%) | 111,531 (73.3%) | **0.141** | -0.067 |
| BMI, mean (SD) | 29.3 (7.2) | 30.3 (6.7) | 29.3 (6.1) | **0.310** | **0.320** |
| BMI, median (IQR) | 28.0 (24.0 – 33.7) | 29.4 (25.8 – 34.2) | 28.6 (25.1 – 32.9) |  |  |
| Smoking status, N (%) |  |  |  |  |  |
| Current | 13,593 (3.5%) | 11,664 (3.4%) | 3,218 (2.1%) | -0.003 | 0.079 |
| Former | 6,490 (1.7%) | 9,494 (2.8%) | 6,483 (4.3%) | 0.076 | -0.081 |
| Never | 35,170 (9.0%) | 26,585 (7.8%) | 12,171 (8.0%) | -0.043 | -0.009 |
| Unknown | 337,394 (85.9%) | 294,696 (86.1%) | 130,302 (85.6%) | 0.004 | 0.012 |

AIDS, acquired immunodeficiency syndrome; BMI, body mass index; ER, emergency room; IQR, interquartile range; HIV, human immunodeficiency virus; OP, outpatient; SD, standard deviation; SMD, standard mean difference

| Supplementary Table 2B: 2016–2017 Season | | | | | |
| --- | --- | --- | --- | --- | --- |
|  | 18–49 Yrs | 50–64 Yrs | ≥65 Yrs | SMD 50–64 vs.  18–49 | SMD 50–64 vs. ≥65 |
|  | N = 1,130,716 | N = 1,003,938 | N = 654,718 |  |  |
| Age, mean (SD) | 35.7 (9.4) | 57.1 (4.2) | 73.6 (6.1) | **7.205** | **-6.148** |
| Age, median (IQR) | 37 (28 – 44) | 57 (54 – 61) | 73 (68 – 79) |  |  |
| Sex, N (%) |  |  |  |  |  |
| Female | 719,706 (63.7%) | 578,766 (57.6%) | 376,989 (57.6%) | **-0.123** | 0.001 |
| Male | 411,010 (36.3%) | 425,172 (42.4%) | 277,729 (42.4%) | **0.123** | -0.001 |
| Race, N (%) |  |  |  |  |  |
| Asian | 35,622 (3.2%) | 25,482 (2.5%) | 15,340 (2.3%) | -0.037 | 0.013 |
| Black or African American | 96,459 (8.5%) | 81,325 (8.1%) | 48,707 (7.4%) | -0.016 | 0.025 |
| Other | 42,332 (3.7%) | 34,020 (3.4%) | 22,477 (3.4%) | -0.019 | -0.002 |
| Unknown | 257,403 (22.8%) | 187,414 (18.7%) | 104,048 (15.9%) | **-0.101** | 0.073 |
| White | 698,900 (61.8%) | 675,697 (67.3%) | 464,146 (70.9%) | **0.115** | -0.078 |
| Ethnicity, N (%) |  |  |  |  |  |
| Hispanic | 110,919 (9.8%) | 70,280 (7.0%) | 45,245 (6.9%) | **-0.101** | 0.004 |
| Non-Hispanic | 935,584 (82.7%) | 862,044 (85.9%) | 569,819 (87.0%) | 0.086 | -0.034 |
| Unknown/not reported | 84,213 (7.4%) | 71,614 (7.1%) | 39,654 (6.1%) | -0.012 | 0.043 |
| Geographic region, N (%) |  |  |  |  |  |
| Northeast | 278,216 (24.6%) | 271,663 (27.1%) | 231,500 (35.4%) | 0.056 | **-0.180** |
| Midwest | 247,229 (21.9%) | 214,486 (21.4%) | 119,471 (18.2%) | -0.012 | 0.078 |
| South | 384,875 (34.0%) | 335,925 (33.5%) | 204,364 (31.2%) | -0.012 | 0.048 |
| West | 219,500 (19.4%) | 180,905 (18.0%) | 98,089 (15.0%) | -0.036 | 0.082 |
| Other/unknown | 896 (0.1%) | 959 (0.1%) | 1,294 (0.2%) | 0.006 | -0.027 |
| Influenza vaccination status, N (%) |  |  |  |  |  |
| No | 937,517 (82.9%) | 744,068 (74.1%) | 424,178 (64.8%) | **-0.215** | **0.204** |
| Yes | 193,199 (17.1%) | 259,870 (25.9%) | 230,540 (35.2%) | **0.215** | **-0.204** |
| Healthcare resource utilization in off-season period |  |  |  |  |  |
| Patients with OP visit, N (%) | 760,547 (67.3%) | 757,741 (75.5%) | 548,063 (83.7%) | **0.182** | **-0.205** |
| Number of all-cause OP visits, mean (SD) | 3.0 (4.0) | 3.3 (4.2) | 3.8 (4.7) | **0.133** | **-0.187** |
| Patients with ER visit, N (%) | 134,977 (11.9%) | 95,757 (9.5%) | 71,804 (11.0%) | -0.078 | -0.047 |
| Number of all-cause ER visits, mean (SD) | 2.0 (3.1) | 2.0 (3.3) | 2.0 (3.2) | 0.001 | -0.022 |
| Patients with hospitalizations, N (%) | 43,341 (3.8%) | 47,185 (4.7%) | 45,224 (6.9%) | 0.043 | -0.095 |
| Number of all-cause hospitalizations, mean (SD) | 1.6 (1.7) | 1.7 (1.8) | 1.5 (1.2) | 0.060 | **0.148** |
| Baseline health status |  |  |  |  |  |
| High-risk patients, N (%) | 408,274 (36.1%) | 586,362 (58.4%) | 507,004 (77.4%) | **0.458** | **-0.416** |
| 1 High risk condition | 206,534 (18.3%) | 252,496 (25.2%) | 158,525 (24.2%) | **0.168** | 0.022 |
| 2 High risk conditions | 115,783 (10.2%) | 162,509 (16.2%) | 137,261 (21.0%) | **0.176** | **-0.123** |
| 3 High risk conditions | 50,199 (4.4%) | 86,976 (8.7%) | 94,537 (14.4%) | **0.171** | **-0.181** |
| 4+ High risk conditions | 35,758 (3.2%) | 84,381 (8.4%) | 116,681 (17.8%) | **0.226** | **-0.282** |
| CCI, mean (SD) | 0.3 (0.9) | 0.9 (1.5) | 1.7 (1.9) | **0.370** | **-0.516** |
| CCI conditions, N (%) |  |  |  |  |  |
| Any malignancy | 14,182 (1.3%) | 52,507 (5.2%) | 75,475 (11.5%) | **0.226** | **-0.229** |
| Congestive heart failure | 10,334 (0.9%) | 35,521 (3.5%) | 68,816 (10.5%) | **0.179** | **-0.275** |
| Cerebrovascular disease | 10,742 (1.0%) | 37,184 (3.7%) | 71,975 (11.0%) | **0.183** | **-0.282** |
| Chronic pulmonary disease | 115,965 (10.3%) | 143,429 (14.3%) | 125,406 (19.2%) | **0.123** | **-0.131** |
| Dementia | 2,414 (0.2%) | 5,970 (0.6%) | 32,452 (5.0%) | 0.060 | -0.268 |
| Diabetes with chronic complication | 16,437 (1.5%) | 55,361 (5.5%) | 82,742 (12.6%) | **0.223** | **-0.250** |
| Diabetes without chronic complication | 78,317 (6.9%) | 191,731 (19.1%) | 199,718 (30.5%) | **0.368** | **-0.266** |
| Hemiplegia or paraplegia | 5,541 (0.5%) | 6,188 (0.6%) | 6,143 (0.9%) | 0.017 | -0.037 |
| Mild liver disease | 29,702 (2.6%) | 51,878 (5.2%) | 28,782 (4.4%) | **0.132** | 0.036 |
| Moderate to severe liver disease | 1,171 (0.1%) | 3,703 (0.4%) | 2,108 (0.3%) | 0.055 | 0.008 |
| Myocardial infarction | 4,792 (0.4%) | 19,857 (2.0%) | 28,645 (4.4%) | **0.143** | **-0.137** |
| Metastatic solid tumor | 1,915 (0.2%) | 6,195 (0.6%) | 6,472 (1.0%) | 0.072 | -0.042 |
| Peripheral vascular disease | 12,000 (1.1%) | 47,778 (4.8%) | 103,243 (15.8%) | **0.221** | **-0.369** |
| Peptic ulcer disease | 5,484 (0.5%) | 9,108 (0.9%) | 9,015 (1.4%) | 0.051 | -0.044 |
| Renal disease, mild to moderate | 11,299 (1.0%) | 33,503 (3.3%) | 71,911 (11.0%) | **0.161** | **-0.300** |
| Severe renal disease | 4,420 (0.4%) | 9,998 (1.0%) | 14,112 (2.2%) | 0.073 | -0.093 |
| Rheumatic disease | 17,715 (1.6%) | 32,128 (3.2%) | 28,169 (4.3%) | 0.107 | -0.058 |
| HIV infection, no AIDS | 4,195 (0.4%) | 4,816 (0.5%) | 926 (0.1%) | 0.017 | 0.061 |
| AIDS | 616 (0.1%) | 700 (0.1%) | 133 (0.0%) | 0.006 | 0.023 |
| Additional High Risk Comorbidities, N (%) |  |  |  |  |  |
| Metabolic disorders | 174,349 (15.4%) | 385,415 (38.4%) | 368,899 (56.3%) | **0.536** | **-0.366** |
| Blood disorders | 79,637 (7.0%) | 102,153 (10.2%) | 125,341 (19.1%) | **0.112** | **-0.256** |
| Neurologic and neurodevelopmental conditions | 13,804 (1.2%) | 3,554 (0.4%) | 1,785 (0.3%) | -0.098 | 0.015 |
| Baseline cardiovascular risk, N (%) |  |  |  |  |  |
| Myocardial infarction-related hospitalizations | 1,144 (0.1%) | 3,957 (0.4%) | 5,154 (0.8%) | 0.059 | -0.051 |
| Ischemic stroke-related hospitalizations | 1,002 (0.1%) | 3,168 (0.3%) | 4,973 (0.8%) | 0.051 | -0.061 |
| Heart failure-related hospitalizations | 2,279 (0.2%) | 8,314 (0.8%) | 16,023 (2.4%) | 0.088 | -0.128 |
| Transient ischemic attack-related hospitalizations | 520 (0.0%) | 1,558 (0.2%) | 2,563 (0.4%) | 0.034 | -0.045 |
| Hypercholesteremia | 195 (0.0%) | 627 (0.1%) | 950 (0.1%) | 0.023 | -0.026 |
| Hypertension | 176,615 (15.6%) | 431,314 (43.0%) | 437,594 (66.8%) | **0.630** | **-0.494** |
| Type 2 diabetes | 73,361 (6.5%) | 191,100 (19.0%) | 203,347 (31.1%) | **0.383** | **-0.280** |
| Systemic lupus erythematosus | 5,848 (0.5%) | 6,193 (0.6%) | 2,916 (0.4%) | 0.013 | 0.024 |
| Rheumatoid arthritis | 11,956 (1.1%) | 24,977 (2.5%) | 21,348 (3.3%) | **0.109** | -0.046 |
| Has BMI, N (%) | 712,043 (63.0%) | 691,842 (68.9%) | 470,983 (71.9%) | **0.126** | -0.066 |
| BMI, mean (SD) | 29.4 (7.3) | 30.3 (6.7) | 29.2 (6.1) | **0.283** | **0.374** |
| BMI, median (IQR) | 28.1 (24.0 – 33.9) | 29.5 (25.8 – 34.2) | 28.4 (25.0 – 32.6) |  |  |
| Smoking status, N (%) |  |  |  |  |  |
| Current | 41,555 (3.7%) | 38,235 (3.8%) | 14,890 (2.3%) | 0.007 | 0.089 |
| Former | 17,874 (1.6%) | 26,273 (2.6%) | 25,280 (3.9%) | 0.072 | -0.070 |
| Never | 82,924 (7.3%) | 65,391 (6.5%) | 43,824 (6.7%) | -0.032 | -0.007 |
| Unknown | 988,363 (87.4%) | 874,039 (87.1%) | 570,724 (87.2%) | -0.010 | -0.003 |

AIDS, acquired immunodeficiency syndrome; BMI, body mass index; ER, emergency room; IQR, interquartile range; HIV, human immunodeficiency virus; OP, outpatient; SD, standard deviation; SMD, standard mean difference

| Supplementary Table 2C: 2017–2018 Season | | | | | |
| --- | --- | --- | --- | --- | --- |
|  | 18–49 Yrs | 50–64 Yrs | ≥65 Yrs | SMD 50–64 vs.  18–49 | SMD 50–64 vs. ≥65 |
|  | N = 1,285,802 | N = 1,129,708 | N = 786,945 |  |  |
| Age, mean (SD) | 35.6 (9.3) | 57.2 (4.2) | 73.8 (6.3) | **7.259** | **-6.111** |
| Age, median (IQR) | 37 (28 – 44) | 57 (54 – 61) | 73 (68 – 79) |  |  |
| Sex, N (%) |  |  |  |  |  |
| Female | 816,181 (63.5%) | 649,522 (57.5%) | 452,568 (57.5%) | **-0.123** | 0.000 |
| Male | 469,621 (36.5%) | 480,186 (42.5%) | 334,377 (42.5%) | **0.123** | 0.000 |
| Race, N (%) |  |  |  |  |  |
| Asian | 43,779 (3.4%) | 31,323 (2.8%) | 19,871 (2.5%) | -0.037 | 0.015 |
| Black or African American | 109,989 (8.6%) | 94,943 (8.4%) | 60,363 (7.7%) | -0.005 | 0.027 |
| Other | 50,618 (3.9%) | 40,960 (3.6%) | 28,154 (3.6%) | -0.016 | 0.003 |
| Unknown | 330,747 (25.7%) | 242,602 (21.5%) | 144,442 (18.4%) | **-0.100** | 0.078 |
| White | 750,669 (58.4%) | 719,880 (63.7%) | 534,115 (67.9%) | **0.110** | -0.088 |
| Ethnicity, N (%) |  |  |  |  |  |
| Hispanic | 130,697 (10.2%) | 86,925 (7.7%) | 57,256 (7.3%) | -0.087 | 0.016 |
| Non-Hispanic | 1,070,926 (83.3%) | 968,336 (85.7%) | 685,953 (87.2%) | 0.067 | -0.042 |
| Unknown/not reported | 84,179 (6.5%) | 74,447 (6.6%) | 43,736 (5.6%) | 0.002 | 0.043 |
| Geographic region, N (%) |  |  |  |  |  |
| Northeast | 321,692 (25.0%) | 315,725 (27.9%) | 272,540 (34.6%) | 0.066 | **-0.145** |
| Midwest | 268,483 (20.9%) | 223,731 (19.8%) | 137,859 (17.5%) | -0.027 | 0.059 |
| South | 430,010 (33.4%) | 373,975 (33.1%) | 251,969 (32.0%) | -0.007 | 0.023 |
| West | 264,576 (20.6%) | 215,114 (19.0%) | 122,582 (15.6%) | -0.039 | 0.092 |
| Other/unknown | 1,041 (0.1%) | 1,163 (0.1%) | 1,995 (0.3%) | 0.007 | -0.036 |
| Influenza vaccination status, N (%) |  |  |  |  |  |
| No | 1,047,227 (81.4%) | 821,879 (72.8%) | 510,004 (64.8%) | **-0.208** | **0.172** |
| Yes | 238,575 (18.6%) | 307,829 (27.2%) | 276,941 (35.2%) | **0.208** | **-0.172** |
| Healthcare resource utilization in off-season period |  |  |  |  |  |
| Patients with OP visit, N (%) | 872,010 (67.8%) | 862,275 (76.3%) | 660,534 (83.9%) | **0.191** | **-0.192** |
| Number of all-cause OP visits, mean (SD) | 3.1 (4.5) | 3.5 (4.5) | 3.9 (4.9) | **0.137** | **-0.174** |
| Patients with ER visit, N (%) | 156,799 (12.2%) | 113,725 (10.1%) | 89,046 (11.3%) | -0.068 | -0.040 |
| Number of all-cause ER visits, mean (SD) | 1.9 (2.9) | 2.0 (3.3) | 2.0 (3.3) | 0.015 | -0.016 |
| Patients with hospitalizations, N (%) | 51,758 (4.0%) | 56,911 (5.0%) | 57,460 (7.3%) | 0.049 | -0.094 |
| Number of all-cause hospitalizations, mean (SD) | 1.7 (1.9) | 1.8 (2.0) | 1.5 (1.4) | 0.076 | **0.156** |
| Baseline health status |  |  |  |  |  |
| High-risk patients, N (%) | 492,585 (38.3%) | 711,789 (63.0%) | 640,643 (81.4%) | **0.510** | **-0.420** |
| 1 High risk condition | 241,276 (18.8%) | 292,995 (25.9%) | 186,867 (23.7%) | **0.173** | 0.051 |
| 2 High risk conditions | 140,051 (10.9%) | 196,602 (17.4%) | 170,341 (21.6%) | **0.188** | **-0.107** |
| 3 High risk conditions | 63,276 (4.9%) | 110,662 (9.8%) | 122,887 (15.6%) | **0.188** | **-0.175** |
| 4+ High risk conditions | 47,982 (3.7%) | 111,530 (9.9%) | 160,548 (20.4%) | **0.246** | **-0.297** |
| CCI, mean (SD) | 0.4 (0.9) | 0.9 (1.5) | 1.8 (2.0) | **0.398** | **-0.529** |
| CCI conditions, N (%) |  |  |  |  |  |
| Any malignancy | 16,594 (1.3%) | 59,978 (5.3%) | 90,684 (11.5%) | **0.226** | **-0.225** |
| Congestive heart failure | 13,203 (1.0%) | 45,119 (4.0%) | 89,842 (11.4%) | **0.191** | **-0.281** |
| Cerebrovascular disease | 13,435 (1.0%) | 46,021 (4.1%) | 91,244 (11.6%) | **0.193** | -0.283 |
| Chronic pulmonary disease | 137,934 (10.7%) | 172,840 (15.3%) | 161,736 (20.6%) | **0.136** | **-0.137** |
| Dementia | 3,518 (0.3%) | 8,093 (0.7%) | 44,273 (5.6%) | 0.063 | -0.283 |
| Diabetes with chronic complication | 24,151 (1.9%) | 80,865 (7.2%) | 121,373 (15.4%) | **0.256** | **-0.263** |
| Diabetes without chronic complication | 96,284 (7.5%) | 232,603 (20.6%) | 250,790 (31.9%) | **0.384** | **-0.259** |
| Hemiplegia or paraplegia | 7,346 (0.6%) | 8,463 (0.7%) | 8,647 (1.1%) | 0.022 | -0.037 |
| Mild liver disease | 39,643 (3.1%) | 66,367 (5.9%) | 40,254 (5.1%) | **0.135** | 0.033 |
| Moderate to severe liver disease | 1,570 (0.1%) | 4,751 (0.4%) | 3,013 (0.4%) | 0.057 | 0.006 |
| Myocardial infarction | 5,845 (0.5%) | 24,259 (2.1%) | 36,005 (4.6%) | **0.150** | **-0.135** |
| Metastatic solid tumor | 2,561 (0.2%) | 7,767 (0.7%) | 8,766 (1.1%) | 0.074 | -0.045 |
| Peripheral vascular disease | 15,686 (1.2%) | 61,180 (5.4%) | 135,199 (17.2%) | **0.236** | **-0.378** |
| Peptic ulcer disease | 6,786 (0.5%) | 11,437 (1.0%) | 11,266 (1.4%) | 0.055 | -0.038 |
| Renal disease, mild to moderate | 14,427 (1.1%) | 42,686 (3.8%) | 94,045 (12.0%) | **0.172** | **-0.307** |
| Severe renal disease | 5,664 (0.4%) | 12,459 (1.1%) | 17,467 (2.2%) | 0.076 | -0.087 |
| Rheumatic disease | 21,768 (1.7%) | 38,473 (3.4%) | 35,916 (4.6%) | **0.109** | -0.059 |
| HIV infection, no AIDS | 4,970 (0.4%) | 5,831 (0.5%) | 1,269 (0.2%) | 0.019 | 0.061 |
| AIDS | 744 (0.1%) | 890 (0.1%) | 192 (0.0%) | 0.008 | 0.024 |
| Additional High Risk Comorbidities, N (%) |  |  |  |  |  |
| Metabolic disorders | 231,967 (18.0%) | 511,141 (45.2%) | 507,645 (64.5%) | **0.612** | **-0.395** |
| Blood disorders | 98,931 (7.7%) | 126,370 (11.2%) | 162,445 (20.6%) | **0.120** | **-0.261** |
| Neurologic and neurodevelopmental conditions | 18,178 (1.4%) | 5,331 (0.5%) | 2,942 (0.4%) | -0.098 | 0.015 |
| Baseline cardiovascular risk, N (%) |  |  |  |  |  |
| Myocardial infarction-related hospitalizations | 1,356 (0.1%) | 5,033 (0.4%) | 6,820 (0.9%) | 0.065 | -0.052 |
| Ischemic stroke-related hospitalizations | 1,291 (0.1%) | 3,917 (0.3%) | 6,435 (0.8%) | 0.052 | -0.062 |
| Heart failure-related hospitalizations | 3,027 (0.2%) | 10,825 (1.0%) | 21,249 (2.7%) | 0.094 | -0.130 |
| Transient ischemic attack-related hospitalizations | 627 (0.0%) | 1,756 (0.2%) | 3,223 (0.4%) | 0.033 | -0.048 |
| Hypercholesteremia | 40,407 (3.1%) | 124,557 (11.0%) | 141,502 (18.0%) | **0.311** | **-0.198** |
| Hypertension | 206,388 (16.1%) | 502,255 (44.5%) | 535,296 (68.0%) | **0.650** | **-0.489** |
| Type 2 diabetes | 91,266 (7.1%) | 234,342 (20.7%) | 258,427 (32.8%) | **0.402** | **-0.276** |
| Systemic lupus erythematosus | 7,297 (0.6%) | 7,577 (0.7%) | 3,859 (0.5%) | 0.013 | 0.024 |
| Rheumatoid arthritis | 14,619 (1.1%) | 29,795 (2.6%) | 27,506 (3.5%) | **0.110** | -0.050 |
| Has BMI, N (%) | 775,034 (60.3%) | 754,251 (66.8%) | 548,464 (69.7%) | **0.135** | -0.063 |
| BMI, mean (SD) | 29.5 (7.3) | 30.4 (6.7) | 29.2 (6.1) | **0.281** | **0.390** |
| BMI, median (IQR) | 28.1 (24.0 – 34.0) | 29.5 (25.8 – 34.3) | 28.3 (25. – 32.7) |  |  |
| Smoking status, N (%) |  |  |  |  |  |
| Current | 48,111 (3.7%) | 45,399 (4.0%) | 18,859 (2.4%) | 0.014 | 0.092 |
| Former | 20,757 (1.6%) | 31,027 (2.7%) | 33,182 (4.2%) | 0.078 | -0.080 |
| Never | 95,004 (7.4%) | 76,080 (6.7%) | 56,015 (7.1%) | -0.026 | -0.015 |
| Unknown | 1,121,930 (87.3%) | 977,202 (86.5%) | 678,889 (86.3%) | -0.022 | 0.007 |

AIDS, acquired immunodeficiency syndrome; BMI, body mass index; ER, emergency room; IQR, interquartile range; HIV, human immunodeficiency virus; OP, outpatient; SD, standard deviation; SMD, standard mean difference

| Supplementary Table 2D: 2018–2019 Season | | | | | |
| --- | --- | --- | --- | --- | --- |
|  | 18–49 Yrs | 50–64 Yrs | ≥65 Yrs | SMD 50–64 vs.  18–49 | SMD 50–64 vs. ≥65 |
|  | N = 1,408,171 | N = 1,249,587 | N = 970,410 |  |  |
| Age, mean (SD) | 35.8 (9.2) | 57.3 (4.2) | 73.8 (6.5) | **7.247** | **-6.044** |
| Age, median (IQR) | 37 (28 – 44) | 57 (54 – 61) | 72 (68 – 79) |  |  |
| Sex, N (%) |  |  |  |  |  |
| Female | 895,206 (63.6%) | 716,458 (57.3%) | 551,393 (56.8%) | **-0.128** | 0.010 |
| Male | 512,965 (36.4%) | 533,129 (42.7%) | 419,017 (43.2%) | **0.128** | -0.010 |
| Race, N (%) |  |  |  |  |  |
| Asian | 45,755 (3.2%) | 34,134 (2.7%) | 24,366 (2.5%) | -0.030 | 0.014 |
| Black or African American | 118,835 (8.4%) | 107,395 (8.6%) | 74,445 (7.7%) | 0.006 | 0.034 |
| Other | 57,523 (4.1%) | 47,462 (3.8%) | 36,448 (3.8%) | -0.015 | 0.002 |
| Unknown | 392,927 (27.9%) | 291,216 (23.3%) | 189,375 (19.5%) | **-0.106** | 0.092 |
| White | 793,131 (56.3%) | 769,380 (61.6%) | 645,776 (66.5%) | **0.107** | **-0.104** |
| Ethnicity, N (%) |  |  |  |  |  |
| Hispanic | 142,660 (10.1%) | 99,397 (8.0%) | 72,393 (7.5%) | -0.076 | 0.019 |
| Non-Hispanic | 1,174,551 (83.4%) | 1,066,693 (85.4%) | 842,306 (86.8%) | 0.054 | -0.041 |
| Unknown/not reported | 90,960 (6.5%) | 83,497 (6.7%) | 55,711 (5.7%) | 0.009 | 0.039 |
| Geographic region, N (%) |  |  |  |  |  |
| Northeast | 314,105 (22.3%) | 310,861 (24.9%) | 280,522 (28.9%) | 0.061 | -0.091 |
| Midwest | 251,670 (17.9%) | 219,050 (17.5%) | 157,137 (16.2%) | -0.009 | 0.036 |
| South | 550,428 (39.1%) | 476,941 (38.2%) | 363,728 (37.5%) | -0.019 | 0.014 |
| West | 291,103 (20.7%) | 241,376 (19.3%) | 165,243 (17.0%) | -0.034 | 0.059 |
| Other/unknown | 865 (0.1%) | 1,359 (0.1%) | 3,780 (0.4%) | 0.016 | -0.056 |
| Influenza vaccination status, N (%) |  |  |  |  |  |
| No | 1,135,553 (80.6%) | 904,247 (72.4%) | 626,609 (64.6%) | **-0.196** | **0.168** |
| Yes | 272,618 (19.4%) | 345,340 (27.6%) | 343,801 (35.4%) | **0.196** | **-0.168** |
| Healthcare resource utilization in off-season period |  |  |  |  |  |
| Patients with OP visit, N (%) | 942,264 (66.9%) | 939,507 (75.2%) | 805,403 (83.0%) | **0.183** | **-0.193** |
| Number of all-cause OP visits, mean (SD) | 3.1 (4.4) | 3.5 (4.6) | 3.9 (4.7) | **0.135** | **-0.160** |
| Patients with ER visit, N (%) | 167,152 (11.9%) | 125,117 (10.0%) | 112,978 (11.6%) | -0.060 | -0.052 |
| Number of all-cause ER visits, mean (SD) | 2.0 (4.0) | 2.1 (4.8) | 2.1 (4.2) | 0.035 | 0.010 |
| Patients with hospitalizations, N (%) | 56,899 (4.0%) | 63,226 (5.1%) | 73,081 (7.5%) | 0.049 | **-0.102** |
| Number of all-cause hospitalizations, mean (SD) | 1.7 (1.9) | 1.8 (2.0) | 1.5 (1.4) | 0.064 | **0.154** |
| Baseline health status |  |  |  |  |  |
| High-risk patients, N (%) | 531,287 (37.7%) | 768,957 (61.5%) | 773,005 (79.7%) | **0.490** | **-0.406** |
| 1 High risk condition | 262,058 (18.6%) | 312,352 (25.0%) | 225,397 (23.2%) | **0.155** | 0.041 |
| 2 High risk conditions | 148,595 (10.6%) | 211,688 (16.9%) | 201,758 (20.8%) | **0.186** | -0.099 |
| 3 High risk conditions | 68,701 (4.9%) | 120,822 (9.7%) | 147,531 (15.2%) | **0.185** | **-0.168** |
| 4+ High risk conditions | 51,933 (3.7%) | 124,095 (9.9%) | 198,319 (20.4%) | **0.250** | **-0.296** |
| CCI, mean (SD) | 0.4 (0.9) | 0.9 (1.5) | 1.8 (2.0) | **0.402** | **-0.521** |
| CCI conditions, N (%) |  |  |  |  |  |
| Any malignancy | 16,145 (1.1%) | 62,292 (5.0%) | 107,918 (11.1%) | **0.224** | **-0.227** |
| Congestive heart failure | 14,532 (1.0%) | 51,205 (4.1%) | 113,625 (11.7%) | **0.195** | **-0.285** |
| Cerebrovascular disease | 14,427 (1.0%) | 51,148 (4.1%) | 110,840 (11.4%) | **0.195** | -0.277 |
| Chronic pulmonary disease | 143,820 (10.2%) | 187,660 (15.0%) | 198,646 (20.5%) | **0.145** | **-0.143** |
| Dementia | 2,696 (0.2%) | 8,182 (0.7%) | 53,273 (5.5%) | 0.071 | **-0.283** |
| Diabetes with chronic complication | 26,086 (1.9%) | 92,627 (7.4%) | 149,417 (15.4%) | **0.267** | **-0.253** |
| Diabetes without chronic complication | 102,609 (7.3%) | 252,742 (20.2%) | 298,412 (30.8%) | **0.382** | **-0.243** |
| Hemiplegia or paraplegia | 7,524 (0.5%) | 9,186 (0.7%) | 10,194 (1.1%) | 0.025 | -0.034 |
| Mild liver disease | 43,764 (3.1%) | 73,637 (5.9%) | 50,265 (5.2%) | **0.135** | **0.031** |
| Moderate to severe liver disease | 1,627 (0.1%) | 4,905 (0.4%) | 3,811 (0.4%) | 0.055 | 0.000 |
| Myocardial infarction | 6,560 (0.5%) | 27,096 (2.2%) | 43,436 (4.5%) | **0.150** | **-0.129** |
| Metastatic solid tumor | 2,415 (0.2%) | 7,989 (0.6%) | 10,711 (1.1%) | 0.074 | -0.050 |
| Peripheral vascular disease | 16,736 (1.2%) | 68,960 (5.5%) | 166,913 (17.2%) | **0.242** | **-0.375** |
| Peptic ulcer disease | 7,219 (0.5%) | 12,316 (1.0%) | 13,978 (1.4%) | 0.055 | -0.042 |
| Renal disease, mild to moderate | 16,094 (1.1%) | 49,215 (3.9%) | 119,090 (12.3%) | **0.178** | **-0.309** |
| Severe renal disease | 6,094 (0.4%) | 13,453 (1.1%) | 20,095 (2.1%) | 0.074 | -0.080 |
| Rheumatic disease | 24,251 (1.7%) | 42,992 (3.4%) | 45,093 (4.6%) | **0.109** | -0.061 |
| HIV infection, no AIDS | 5,182 (0.4%) | 6,570 (0.5%) | 1,735 (0.2%) | 0.024 | 0.059 |
| AIDS | 761 (0.1%) | 976 (0.1%) | 266 (0.0%) | 0.009 | 0.022 |
| Additional High Risk Comorbidities, N (%) |  |  |  |  |  |
| Metabolic disorders | 251,478 (17.9%) | 551,514 (44.1%) | 608,710 (62.7%) | **0.593** | **-0.379** |
| Blood disorders | 109,928 (7.8%) | 140,678 (11.3%) | 203,115 (20.9%) | **0.118** | **-0.266** |
| Neurologic and neurodevelopmental conditions | 18,313 (1.3%) | 4,920 (0.4%) | 2,826 (0.3%) | -0.099 | 0.018 |
| Baseline cardiovascular risk, N (%) |  |  |  |  |  |
| Myocardial infarction-related hospitalizations | 1,661 (0.1%) | 5,800 (0.5%) | 8,591 (0.9%) | 0.064 | -0.051 |
| Ischemic stroke-related hospitalizations | 1,539 (0.1%) | 4,827 (0.4%) | 8,215 (0.8%) | 0.056 | -0.059 |
| Heart failure-related hospitalizations | 3,692 (0.3%) | 12,934 (1.0%) | 28,374 (2.9%) | 0.096 | -0.136 |
| Transient ischemic attack-related hospitalizations | 596 (0.0%) | 1,947 (0.2%) | 3,656 (0.4%) | 0.036 | -0.043 |
| Hypercholesteremia | 42,459 (3.0%) | 130,841 (10.5%) | 163,709 (16.9%) | **0.301** | **-0.187** |
| Hypertension | 220,981 (15.7%) | 543,151 (43.5%) | 641,492 (66.1%) | **0.639** | **-0.467** |
| Type 2 diabetes | 96,631 (6.9%) | 254,843 (20.4%) | 308,010 (31.7%) | **0.402** | **-0.261** |
| Systemic lupus erythematosus | 8,195 (0.6%) | 8,357 (0.7%) | 4,835 (0.5%) | 0.011 | 0.022 |
| Rheumatoid arthritis | 16,269 (1.2%) | 33,411 (2.7%) | 34,916 (3.6%) | **0.111** | -0.053 |
| Has BMI, N (%) | 845,095 (60.0%) | 833,937 (66.7%) | 680,453 (70.1%) | **0.140** | -0.073 |
| BMI, mean (SD) | 29.7 (7.4) | 30.5 (6.8) | 29.1 (6.2) | **0.258** | **0.441** |
| BMI, median (IQR) | 28.3 (24 – 34) | 29.7 (25.9 – 34.4) | 28.2 (25.0 – 32.6) |  |  |
| Smoking status, N (%) |  |  |  |  |  |
| Current | 56,592 (4.0%) | 54,772 (4.4%) | 25,785 (2.7%) | 0.018 | 0.094 |
| Former | 22,924 (1.6%) | 34,069 (2.7%) | 41,788 (4.3%) | 0.075 | -0.086 |
| Never | 109,141 (7.8%) | 89,040 (7.1%) | 74,345 (7.7%) | -0.024 | -0.020 |
| Unknown | 1,219,514 (86.6%) | 1,071,706 (85.8%) | 828,492 (85.4%) | -0.024 | 0.011 |

AIDS, acquired immunodeficiency syndrome; BMI, body mass index; ER, emergency room; IQR, interquartile range; HIV, human immunodeficiency virus; OP, outpatient; SD, standard deviation; SMD, standard mean difference

| Supplementary Table 2E: 2019–2020 Season | | | | | |
| --- | --- | --- | --- | --- | --- |
|  | 18–49 Yrs | 50–64 Yrs | ≥65 Yrs | SMD 50–64 vs.  18–49 | SMD 50–64 vs. ≥65 |
|  | N = 1,252,459 | N = 1,108,895 | N = 949,582 |  |  |
| Age, mean (SD) | 35.8 (9.2) | 57.3 (4.2) | 74.0 (6.7) | **7.266** | **-6.047** |
| Age, median (IQR) | 37 (29 – 44) | 58 (54 – 61) | 73 (68 – 79) |  |  |
| Sex, N (%) |  |  |  |  |  |
| Female | 793,082 (63.3%) | 631,525 (57.0%) | 534,725 (56.3%) | **-0.130** | 0.013 |
| Male | 459,377 (36.7%) | 477,370 (43.0%) | 414,857 (43.7%) | **0.130** | -0.013 |
| Race, N (%) |  |  |  |  |  |
| Asian | 40,473 (3.2%) | 30,132 (2.7%) | 22,870 (2.4%) | -0.030 | 0.020 |
| Black or African American | 99,899 (8.0%) | 91,212 (8.2%) | 68,369 (7.2%) | 0.009 | 0.038 |
| Other | 51,973 (4.1%) | 43,119 (3.9%) | 35,896 (3.8%) | -0.013 | 0.006 |
| Unknown | 375,256 (30.0%) | 277,078 (25.0%) | 196,517 (20.7%) | **-0.112** | **0.102** |
| White | 684,858 (54.7%) | 667,354 (60.2%) | 625,930 (65.9%) | **0.111** | **-0.119** |
| Ethnicity, N (%) |  |  |  |  |  |
| Hispanic | 120,864 (9.7%) | 88,842 (8.0%) | 70,471 (7.4%) | -0.058 | 0.022 |
| Non-Hispanic | 1,050,450 (83.9%) | 944,367 (85.2%) | 823,911 (86.8%) | 0.036 | -0.046 |
| Unknown/not reported | 81,145 (6.5%) | 75,686 (6.8%) | 55,200 (5.8%) | 0.014 | 0.042 |
| Geographic region, N (%) |  |  |  |  |  |
| Northeast | 282,528 (22.6%) | 280,434 (25.3%) | 264,321 (27.8%) | 0.064 | -0.058 |
| Midwest | 193,875 (15.5%) | 165,714 (14.9%) | 140,746 (14.8%) | -0.015 | 0.003 |
| South | 514,409 (41.1%) | 445,452 (40.2%) | 376,151 (39.6%) | -0.018 | 0.011 |
| West | 261,388 (20.9%) | 216,331 (19.5%) | 163,484 (17.2%) | -0.034 | 0.059 |
| Other/unknown | 259 (0.0%) | 964 (0.1%) | 4,880 (0.5%) | 0.029 | -0.078 |
| Influenza vaccination status, N (%) |  |  |  |  |  |
| No | 986,340 (78.8%) | 787,115 (71.0%) | 608,510 (64.1%) | **-0.180** | **0.148** |
| Yes | 266,119 (21.2%) | 321,780 (29.0%) | 341,072 (35.9%) | **0.180** | **-0.148** |
| Healthcare resource utilization in off-season period |  |  |  |  |  |
| Patients with OP visit, N (%) | 852,986 (68.1%) | 841,232 (75.9%) | 788,746 (83.1%) | **0.173** | **-0.179** |
| Number of all-cause OP visits, mean (SD) | 3.1 (4.0) | 3.5 (4.0) | 3.9 (4.5) | **0.130** | **-0.185** |
| Patients with ER visit, N (%) | 146,313 (11.7%) | 110,223 (9.9%) | 112,395 (11.8%) | -0.056 | -0.061 |
| Number of all-cause ER visits, mean (SD) | 1.9 (2.8) | 2.0 (3.2) | 2.0 (3.0) | 0.026 | -0.021 |
| Patients with hospitalizations, N (%) | 48,720 (3.9%) | 55,149 (5.0%) | 73,790 (7.8%) | 0.053 | **-0.115** |
| Number of all-cause hospitalizations, mean (SD) | 1.6 (1.8) | 1.8 (1.9) | 1.5 (1.4) | 0.062 | **0.140** |
| Baseline health status |  |  |  |  |  |
| High-risk patients, N (%) | 475,259 (37.9%) | 684,762 (61.8%) | 761,366 (80.2%) | **0.490** | **-0.415** |
| 1 High risk condition | 234,064 (18.7%) | 276,864 (25.0%) | 216,416 (22.8%) | **0.152** | 0.051 |
| 2 High risk conditions | 132,366 (10.6%) | 187,149 (16.9%) | 196,163 (20.7%) | **0.184** | -0.097 |
| 3 High risk conditions | 61,471 (4.9%) | 108,366 (9.8%) | 145,697 (15.3%) | **0.187** | **-0.169** |
| 4+ High risk conditions | 47,358 (3.8%) | 112,383 (10.1%) | 203,090 (21.4%) | **0.252** | **-0.313** |
| CCI, mean (SD) | 0.4 (0.9) | 0.9 (1.6) | 1.8 (2.1) | **0.406** | **-0.542** |
| CCI conditions, N (%) |  |  |  |  |  |
| Any malignancy | 14,471 (1.2%) | 56,224 (5.1%) | 108,272 (11.4%) | **0.227** | **-0.232** |
| Congestive heart failure | 12,393 (1.0%) | 45,248 (4.1%) | 115,234 (12.1%) | **0.198** | **-0.298** |
| Cerebrovascular disease | 13,153 (1.1%) | 46,767 (4.2%) | 110,557 (11.6%) | **0.199** | **-0.277** |
| Chronic pulmonary disease | 125,170 (10.0%) | 162,420 (14.6%) | 193,531 (20.4%) | **0.142** | **-0.151** |
| Dementia | 2,333 (0.2%) | 7,304 (0.7%) | 55,721 (5.9%) | **0.073** | **-0.296** |
| Diabetes with chronic complication | 23,096 (1.8%) | 83,631 (7.5%) | 151,253 (15.9%) | **0.272** | **-0.263** |
| Diabetes without chronic complication | 90,240 (7.2%) | 222,563 (20.1%) | 291,362 (30.7%) | **0.382** | **-0.246** |
| Hemiplegia or paraplegia | 6,386 (0.5%) | 7,991 (0.7%) | 10,080 (1.1%) | 0.027 | -0.036 |
| Mild liver disease | 41,372 (3.3%) | 67,468 (6.1%) | 52,426 (5.5%) | **0.132** | 0.024 |
| Moderate to severe liver disease | 1,514 (0.1%) | 4,679 (0.4%) | 4,103 (0.4%) | 0.058 | -0.002 |
| Myocardial infarction | 5,881 (0.5%) | 24,360 (2.2%) | 42,367 (4.5%) | **0.151** | **-0.127** |
| Metastatic solid tumor | 2,340 (0.2%) | 7,611 (0.7%) | 11,765 (1.2%) | 0.076 | -0.057 |
| Peripheral vascular disease | 15,103 (1.2%) | 63,130 (5.7%) | 172,971 (18.2%) | **0.248** | **-0.393** |
| Peptic ulcer disease | 6,582 (0.5%) | 10,997 (1.0%) | 13,731 (1.4%) | 0.054 | -0.041 |
| Renal disease, mild to moderate | 14,500 (1.2%) | 44,949 (4.1%) | 122,526 (12.9%) | **0.183** | **-0.322** |
| Severe renal disease | 5,192 (0.4%) | 11,946 (1.1%) | 19,690 (2.1%) | 0.077 | -0.080 |
| Rheumatic disease | 22,134 (1.8%) | 39,428 (3.6%) | 45,704 (4.8%) | **0.111** | -0.063 |
| HIV infection, no AIDS | 4,779 (0.4%) | 6,231 (0.6%) | 1,724 (0.2%) | 0.026 | 0.063 |
| AIDS | 620 (0.0%) | 919 (0.1%) | 249 (0.0%) | 0.013 | 0.024 |
| Additional High Risk Comorbidities, N (%) |  |  |  |  |  |
| Metabolic disorders | 232,650 (18.6%) | 500,672 (45.2%) | 609,453 (64.2%) | **0.595** | **-0.389** |
| Blood disorders | 98,614 (7.9%) | 127,018 (11.5%) | 207,387 (21.8%) | **0.121** | **-0.282** |
| Neurologic and neurodevelopmental conditions | 15,923 (1.3%) | 4,133 (0.4%) | 2,602 (0.3%) | -0.100 | 0.017 |
| Baseline cardiovascular risk, N (%) |  |  |  |  |  |
| Myocardial infarction-related hospitalizations | 1,494 (0.1%) | 5,414 (0.5%) | 8,684 (0.9%) | 0.067 | -0.051 |
| Ischemic stroke-related hospitalizations | 1,364 (0.1%) | 4,321 (0.4%) | 8,326 (0.9%) | 0.056 | -0.061 |
| Heart failure-related hospitalizations | 3,203 (0.3%) | 12,023 (1.1%) | 28,792 (3.0%) | **0.102** | **-0.138** |
| Transient ischemic attack-related hospitalizations | 515 (0.0%) | 1,608 (0.1%) | 3,255 (0.3%) | 0.034 | -0.040 |
| Hypercholesteremia | 37,759 (3.0%) | 113,711 (10.3%) | 154,533 (16.3%) | **0.294** | **-0.178** |
| Hypertension | 194,834 (15.6%) | 476,759 (43.0%) | 626,191 (65.9%) | **0.632** | **-0.474** |
| Type 2 diabetes | 84,624 (6.8%) | 224,385 (20.2%) | 302,211 (31.8%) | **0.402** | **-0.266** |
| Systemic lupus erythematosus | 7,561 (0.6%) | 7,822 (0.7%) | 4,930 (0.5%) | 0.013 | 0.024 |
| Rheumatoid arthritis | 14,853 (1.2%) | 30,505 (2.8%) | 35,560 (3.7%) | **0.113** | -0.056 |
| Has BMI, N (%) | 764,074 (61.0%) | 753,797 (68.0%) | 675,337 (71.1%) | **0.146** | -0.068 |
| BMI, mean (SD) | 29.7 (7.4) | 30.5 (6.8) | 29.1 (6.2) | **0.261** | **0.458** |
| BMI, median (IQR) | 28.4 (24.0 – 34.0) | 29.7 (26.0 – 34.5) | 28.2 (25.0 – 32.6) |  |  |
| Smoking status, N (%) |  |  |  |  |  |
| Current | 50,747 (4.1%) | 49,161 (4.4%) | 26,568 (2.8%) | 0.019 | 0.088 |
| Former | 20,921 (1.7%) | 31,207 (2.8%) | 43,544 (4.6%) | 0.077 | -0.094 |
| Never | 55,824 (4.5%) | 44,706 (4.0%) | 38,697 (4.1%) | -0.021 | -0.002 |
| Unknown | 1,124,967 (89.8%) | 983,821 (88.7%) | 840,773 (88.5%) | -0.036 | 0.006 |

AIDS, acquired immunodeficiency syndrome; BMI, body mass index; ER, emergency room; IQR, interquartile range; HIV, human immunodeficiency virus; OP, outpatient; SD, standard deviation; SMD, standard mean difference
